# Pseudomonas aeruginosa Promotes Persistence of Stenotrophomonas maltophilia via Increased Adherence to Depolarized Respiratory Epithelium

**DOI:** 10.1128/spectrum.03846-22

**Published:** 2022-12-06

**Authors:** Melissa S. McDaniel, Natalie R. Lindgren, Caitlin E. Billiot, Kristina N. Valladares, Nicholas A. Sumpter, W. Edward Swords

**Affiliations:** a Division of Pulmonary, Allergy and Critical Care Medicine, University of Alabama at Birmingham, Birmingham, Alabama, USA; b Gregory Fleming James Center for Cystic Fibrosis Research, University of Alabama at Birmingham, Birmingham, Alabama, USA; c Division of Clinical Immunology and Rheumatology, University of Alabama at Birmingham, Birmingham, Alabama, USA; Griffith University

**Keywords:** *Stenotrophomonas maltophilia*, *Pseudomonas aeruginosa*, polymicrobial infection, type IV pilus, adherence, respiratory epithelium, pilus, cystic fibrosis

## Abstract

Stenotrophomonas maltophilia is an emerging opportunistic respiratory pathogen in people with cystic fibrosis (CF). S. maltophilia is frequently observed in polymicrobial infections, and we have previously shown that Pseudomonas aeruginosa promotes colonization and persistence of S. maltophilia in mouse respiratory infections. In this study, we used host and bacterial RNA sequencing to further understand the molecular underpinnings of this interaction. To evaluate S. maltophilia transcript profiles, we used a recently described method for selective capture of bacterial mRNA transcripts with strain-specific RNA probes. We found that factors associated with the type IV pilus, including the histidine kinase subunit of a chemotactic two-component signaling system (*chpA*), had increased transcript levels during dual-species infection. Using immortalized CF respiratory epithelial cells, we found that infection with P. aeruginosa increases adherence of S. maltophilia, at least in part due to disruption of epithelial tight junctions. In contrast, an isogenic S. maltophilia *chpA* mutant strain lacked cooperative adherence to CF epithelia and decreased bacterial burden *in vivo* in dual-species infections with P. aeruginosa. Similarly, P. aeruginosa lacking elastase (*lasB*) failed to promote S. maltophilia adherence or bacterial colonization and persistence *in vivo*. Based on these results, we propose that disruption of lung tissue integrity by P. aeruginosa facilitates adherence of S. maltophilia to the lung epithelia, likely in a type IV pilus-dependent manner. These data lend insight into S. maltophilia colonization and persistence in people in later stages of CF disease and may have implications for interactions with other bacterial opportunists.

**IMPORTANCE** Despite advances in treatment options for people with CF, complications of bacterial infections remain the greatest driver of morbidity and mortality in this patient population. These infections often involve more than one bacterial pathogen, and our understanding of how interspecies interactions impact disease progression is lacking. Previous work in our lab found that two CF pathogens, Stenotrophomonas maltophilia and Pseudomonas aeruginosa, can work together in the lung to cause more severe infection. In the present study, we found that infection with P. aeruginosa promotes persistence of S. maltophilia by interfering with epithelial barrier integrity. Depolarization of the epithelial cell layer by P. aeruginosa-secreted elastase increased S. maltophilia adherence, likely in a type IV pilus-dependent manner. Ultimately, this work sheds light on the molecular mechanisms governing an important multispecies interaction seen in pulmonary diseases such as CF.

## INTRODUCTION

Stenotrophomonas maltophilia is a Gram-negative bacillus that can be found in a variety of environmental sources, including in hospital tubing and water systems (1–4). While S. maltophilia acts as an opportunistic pathogen in many disease contexts, including bloodstream infections in those immunocompromised due to cancer, however, it is most commonly associated with pulmonary infections (5–8). This includes acute infections such as ventilator-associated pneumonia (VAP) and chronic airway diseases like cystic fibrosis (CF) (9–12). In the context of CF, the presence of S. maltophilia in patient sputa has been correlated with worse lung function (13, 14). Whole-genome sequencing of S. maltophilia has revealed homologs of many known virulence factors, including fimbriae, flagella, and type IV pili (15). There is a pressing need for a better definition of factors involved in colonization, persistence, and/or virulence of S. maltophilia.

Pseudomonas aeruginosa is a Gram-negative bacillus that, like S. maltophilia, can be found in a variety of environmental contexts. It is an opportunistic pathogen, primarily affecting those with an underlying immunodeficiency or disease, and is a common opportunist observed in those with CF, where it contributes significantly to morbidity and mortality (12). P. aeruginosa has a relatively large genome (~6.5 Mb), harboring many virulence factors that have been identified and characterized (16). Importantly, P. aeruginosa can secrete a number of toxins and extracellular proteases, notably ExoA, elastase, and pyocyanin, that can contribute to lung function decline and work synergistically to compromise airway barrier integrity (17).

In chronic lung diseases such as CF, infections are often polymicrobial, and interspecies dynamics can play a large role in patient outcomes. Reports indicate that P. aeruginosa can be coisolated with S. maltophilia out of the lung from people with CF, VAP, and, more recently, hospital-acquired pneumonia in those hospitalized for COVID-19 (18–21). Several *in vitro* studies have shown mechanisms of cooperativity between S. maltophilia and P. aeruginosa, including changes in antibiotic tolerance and biofilm formation by S. maltophilia and increased alginate and toxin production by P. aeruginosa (22, 23). In previous work, we demonstrated cooperativity between P. aeruginosa and S. maltophilia during dual-species infection in the mouse respiratory tract (24). In this study, intratracheal infection with S. maltophilia and P. aeruginosa resulted in a significantly higher bacterial burden of S. maltophilia in lung homogenate and a longer time to clearance than mice infected with S. maltophilia alone.

In this study, we sought to understand the mechanism by which P. aeruginosa promotes colonization with S. maltophilia. We used combined bacterial and host RNA sequencing from murine pulmonary infections with *in vitro* adherence assays on polarized epithelium to elucidate the systems involved in cooperativity between S. maltophilia and P. aeruginosa. The results indicate that damage to the airway epithelium mediated by P. aeruginosa allows for increased binding by S. maltophilia, likely via the type IV pilus.

## RESULTS

### Host response to single- and dual-species infection is dominated by P. aeruginosa-induced inflammatory response.

**FIG 1:**
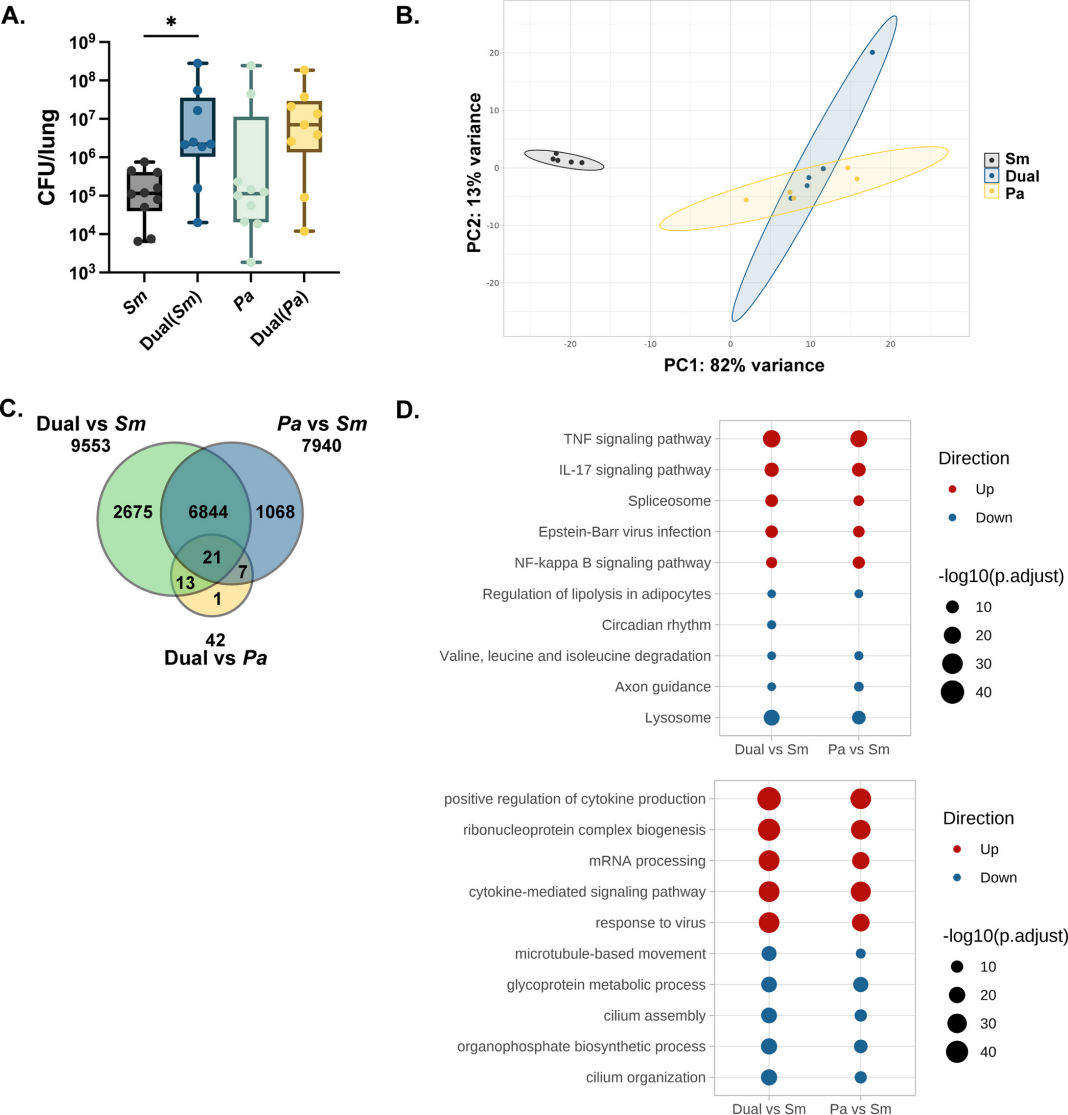
The host inflammatory response following dual-species infection is driven by P. aeruginosa. BALB/cJ mice were intratracheally infected with ~10^7^ CFU of S. maltophilia K279a and P. aeruginosa mPA08-31 alone and in combination. Groups were euthanized at 24 h postinfection. (A) Bacterial load in lung homogenate was enumerated via viable colony counting on differential medium (median ± interquartile range [IQR]; *n* = 9 to 10; Kruskal-Wallis with Dunn’s multiple comparisons; *, *P* < 0.05). Using the same infection scheme, whole-lung RNA was extracted and sequenced (*n* = 5). Pa, P. aeruginosa; Sm, S. maltophilia. (B) Principal-component analysis of samples based on RNA sequencing data, colored by infection group. (C) Venn diagram depicting the number of significantly differentially expressed genes between groups as determined via differential expression analysis using DESeq2. (D) Pathway analysis of differentially expressed genes performed via clusterProfiler using KEGG pathway (left) and Gene Ontology (biological function) (right) and pathway databases (right). The top 10 differentially regulated pathways (5 most enriched by positively expressed genes and 5 most enriched by negatively expressed genes) are represented for each comparison.

Our recent work showed a cooperative interaction between two CF pathogens, S. maltophilia and P. aeruginosa, during murine pulmonary infection where the presence of P. aeruginosa promotes the persistence of S. maltophilia (Fig. 1A) (24). In order to define the basis for this cooperativity, we first performed host RNA sequencing analysis (RNA-seq) on whole lung from mice with single- or dual-species infections. Mice were infected intratracheally with S. maltophilia K279a, the type strain of S. maltophilia for which the genome is well characterized (inoculum, ~10^7^ CFU); P. aeruginosa mPA08-31, a mucoid clinical isolate derived from the sputum of a patient with CF (inoculum ~10^7^ CFU); or both before total RNA was collected from the lung. The RNA was then prepared for sequencing and sequenced at a depth of ~30 million reads per sample.

Principal-component analysis of mouse gene expression data showed close clustering of P. aeruginosa and dual species-infected samples, while S. maltophilia-infected animals clustered separately from both (Fig. 1B). Differential expression analysis between samples showed that 9,553 genes are differentially regulated between dual species-infected mice and mice infected with S. maltophilia alone. Similarly, 7,940 genes are differentially regulated between mice infected with P. aeruginosa alone and mice infected with S. maltophilia alone. Consistent with the principal-component analysis, only 42 genes were differentially regulated between mice infected with P. aeruginosa alone and dual species-infected mice. Of the 9,553 differentially expressed genes between dual species-infected mice and mice infected with S. maltophilia alone, 6,844 were also differentially expressed between mice infected with P. aeruginosa alone and mice infected with S. maltophilia alone. Only 21 genes were differentially expressed in all three comparisons (Fig. 1C).

To determine which biological processes or pathways were affected during infection, we performed pathway enrichment analysis on the list of differentially expressed genes from each comparison. This was performed using ClusterProfiler (25) with both Gene Ontology (GO) biological processes and Kyoto Encyclopedia of Genes and Genomes (KEGG) pathway databases (Fig. 1D). Upregulated genes from the dual-species and P. aeruginosa infections compared to S. maltophilia infection were enriched for a total of 2,206 unique GO terms (1,952 and 1,918, respectively) and 81 unique KEGG pathways (75 and 68, respectively). The 5 most enriched GO terms among upregulated genes included positive regulation of cytokine production (*P*_adj_, 6.71 × 10^−46^ and 7.81 × 10^−39^, respectively) and cytokine-mediated signaling pathways (*P*_adj_, 1.12 × 10^−40^ and 1.76 × 10^−36^, respectively). Of the 5 most enriched KEGG pathways implicated by upregulated genes, we identified known proinflammatory pathways, including tumor necrosis factor (TNF) (*P*_adj_, 2.99 × 10^−21^ and 4.02 × 10^−20^, respectively) and interleukin 17 (IL-17) signaling (*P*_adj_, 9.03 × 10^−13^ and 8.79 × 10^−13^, respectively). The enrichment of these biological processes is consistent with an increase in acute inflammatory response and lung damage during P. aeruginosa infection (26). Downregulated genes in these comparisons were enriched for 1,321 unique GO terms (1,108 and 1,073, respectively) and 50 unique KEGG pathways (42 and 32, respectively). Interestingly, both cilium organization and cilium assembly processes were among the 5 most enriched GO terms for downregulated genes (*P*_adj_, 1.89 × 10^−20^ and 6.45 × 10^−14^, respectively), indicating possible disruption of the mucociliary clearance mechanism (27). The 5 most enriched KEGG pathways among downregulated genes highlighted many metabolic processes, including amino acid degradation and fatty acid metabolism.

### Dual-species infection increases expression of adherence and chemotaxis-related genes in S. maltophilia.

Traditionally, RNA sequencing of pathogen transcripts in the lung has been difficult due to the overwhelming proportion of host RNA compared to bacterial RNA. To circumvent this, we employed a previously published method for selective hybridization and capture of bacterial mRNA, previously named pathogen-hybrid capture (PatH-Cap) (28). Strain-specific RNA probe libraries are used to capture pathogen-specific transcripts of interest, allowing for enrichment of bacterial mRNA transcripts and sequencing of the pathogen transcriptome with sufficient coverage, even in the context of RNA extracted from host tissue (see Fig. S1 in the supplemental material). For the samples from mice infected with S. maltophilia in the absence of P. aeruginosa, transcript capture resulted in a 697-fold increase in reads mapping to the S. maltophilia transcriptome (from 0.01% prior to enrichment to 6.97% postenrichment). For those samples from mice infected with S. maltophilia in the presence of P. aeruginosa, this increase was 770-fold (from 0.10% to 77.01%) (Table S1).

**FIG 2:**
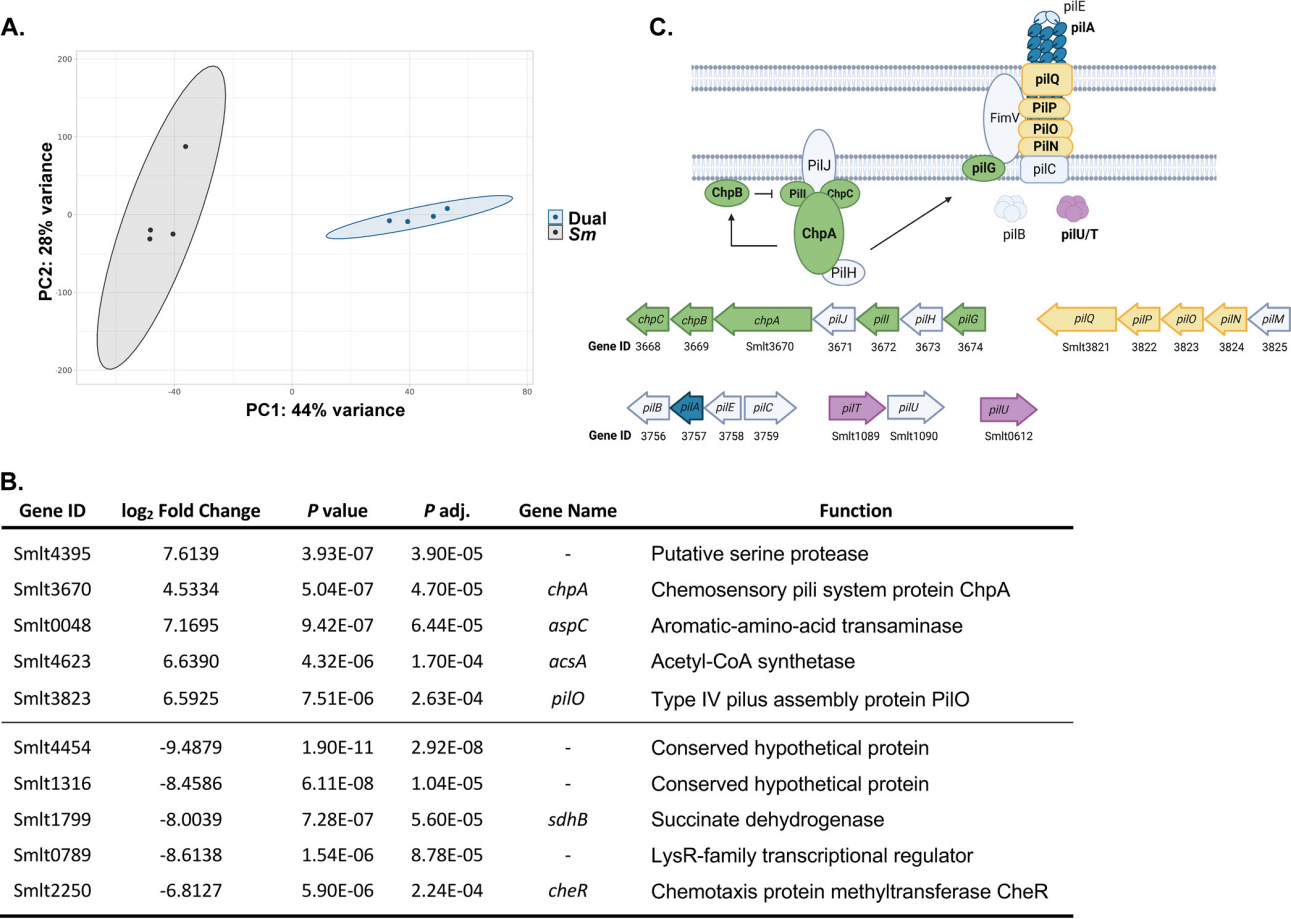
S. maltophilia upregulates genes associated with adhesion and chemotaxis in the context of dual-species infection. Pathogen-enriched RNA was extracted as detailed above and sequenced (*n* = 4). (A) Principal-component analysis of samples based on bacterium-specific RNA sequencing data, colored by infection group. (B) Top 5 most significantly up- and downregulated S. maltophilia genes during coinfection in the lung compared to single-species infection, determined via DESeq2. Genes with reads detected in less than half of the samples for each group were excluded from this analysis. *P* adj. indicates the significance value after multiple testing corrections. (C) Schematic depicting the proposed type IV pilus system of S. maltophilia (31, 55, 56). Loci are represented as annotated in S. maltophilia K279a (15). Genes significantly upregulated during dual-species infection are highlighted in color for each locus.

Principal-component analysis of selective capture-enriched S. maltophilia transcript data showed distinct clustering between samples from mice infected with S. maltophilia alone and dual species-infected samples (Fig. 2A). Differential expression analysis between samples showed 686 S. maltophilia genes that are differentially regulated (*P*_adj_ < 0.05) between these two conditions. To account for disparities in genome coverage between sample groups, we filtered results for genes with detectable transcripts in 2 out of 4 total samples in each group, resulting in a total of 149 differentially expressed genes. Of these, the top 5 significantly upregulated genes included a previously uncharacterized serine protease (*Smlt4395*, *P*_adj_, 3.90 × 10^−5^) and 2 genes involved in type IV pilus biogenesis or regulation, *chpA* (*Smlt3670*, *P*_adj_, 4.70 × 10^−5^) and *pilO* (*Smlt3823*, *P*_adj_, 2.63 × 10^−4^) (29–31). Interestingly, one of the most significantly downregulated genes during dual-species infection was *cheR* (*Smlt2250*, *P*_adj_, 2.24 × 10^−4^), a determinant in the regulation of flagellar movement (32), indicating that motility and attachment processes are changing in the context of dual-species infection (Fig. 2B). In support of this, of the 19 genes in 3 of the operons predicted to govern type IV pilus biogenesis and regulation in S. maltophilia, 12 genes (shown in color) were significantly upregulated during dual-species infection in the lung (Fig. 2C; Table S2). This was not the case for genes involved in fimbriae or flagella regulation and biogenesis. Only 1 out of 47 total predicted flagella-related genes was significantly upregulated during dual-species infection, and there was no significant change for *smf-1*, the major protein involved in fimbrial function (Table S3).

### Preinfection with P. aeruginosa increases adherence of S. maltophilia to a polarized epithelium.

**FIG 3:**
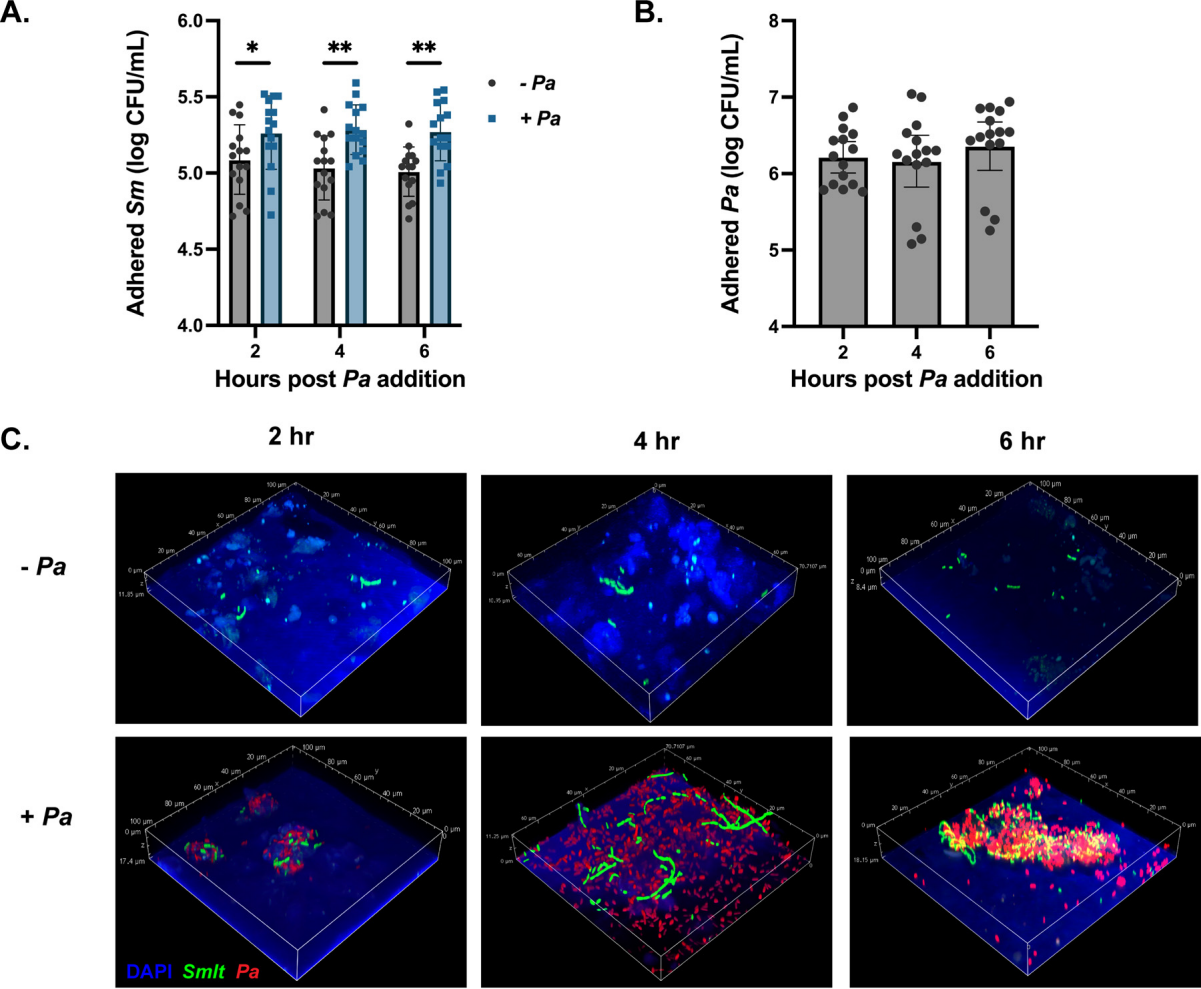
Preexposure of epithelial cells to P. aeruginosa promotes adherence of S. maltophilia. Immortalized cystic fibrosis bronchial epithelial cells (CFBEs) were grown at the air-liquid interface until they were polarized. Cells were pretreated with either minimum essential medium with Earle’s salts (EMEM) or ~10^6^ CFU of P. aeruginosa mPA08-31 for 2, 4, or 6 h before the addition of ~10^6^ S. maltophilia K279a for 1 h. (A and B) Viable colony counts of adherent S. maltophilia K279a (geometric mean ± geometric standard deviation [GSD]; *n* = 15 wells; two-way ANOVA; *, *P* < 0.05; **, *P* < 0.01) (A) or P. aeruginosa mPA08-31 (geometric mean ± GSD, *n* = 15 wells) (B). (C) Structural composition of dual-species foci as evaluated via confocal microscopy. Infections were repeated with S. maltophilia K279a (gfp^+^) and P. aeruginosa mPA08-31 (mCherry^+^), and CFBEs were visualized with DAPI. Dual-species foci were imaged at ×60 magnification.

The increased expression of genes involved in bacterial chemotaxis and adherence, combined with previous reports that exposure of epithelial cells to P. aeruginosa can promote S. maltophilia adherence (33), prompted us to investigate whether this was a viable mechanism for microbial cooperativity in the lung. To do this, we first polarized immortalized cystic fibrosis bronchial epithelial cells (CFBEs) by culturing them at the air-liquid interface. We then pretreated the polarized epithelia with P. aeruginosa mPA08-31 (multiplicity of infection [MOI] = 20) for 2, 4, or 6 h prior to inoculation with S. maltophilia K279a (MOI = 20) for 1 h. Following this, we evaluated adherence efficiency via viable colony counts and confocal microscopy (Fig. 3A). We found that pretreatment of cells with P. aeruginosa significantly increased the number of adherent S. maltophilia (*P* < 0.0001), with the largest difference between treated and untreated cells occurring at 6 h postinfection (Fig. 3B). This coincides with the time point at which the burden of P. aeruginosa is the highest (Fig. 3C). Imaging of infected cells via confocal scanning laser microscopy (CSLM) showed more S. maltophilia present on cells pretreated with P. aeruginosa than on those exposed to cell culture media alone at all time points. We also found that S. maltophilia bound to epithelial cells near P. aeruginosa, with the largest foci of both bacteria present in cells pretreated for 6 h (Fig. 3C).

### Pretreatment with P. aeruginosa promotes binding of S. maltophilia to a polarized epithelium in a *chpA*-dependent manner.

**FIG 4:**
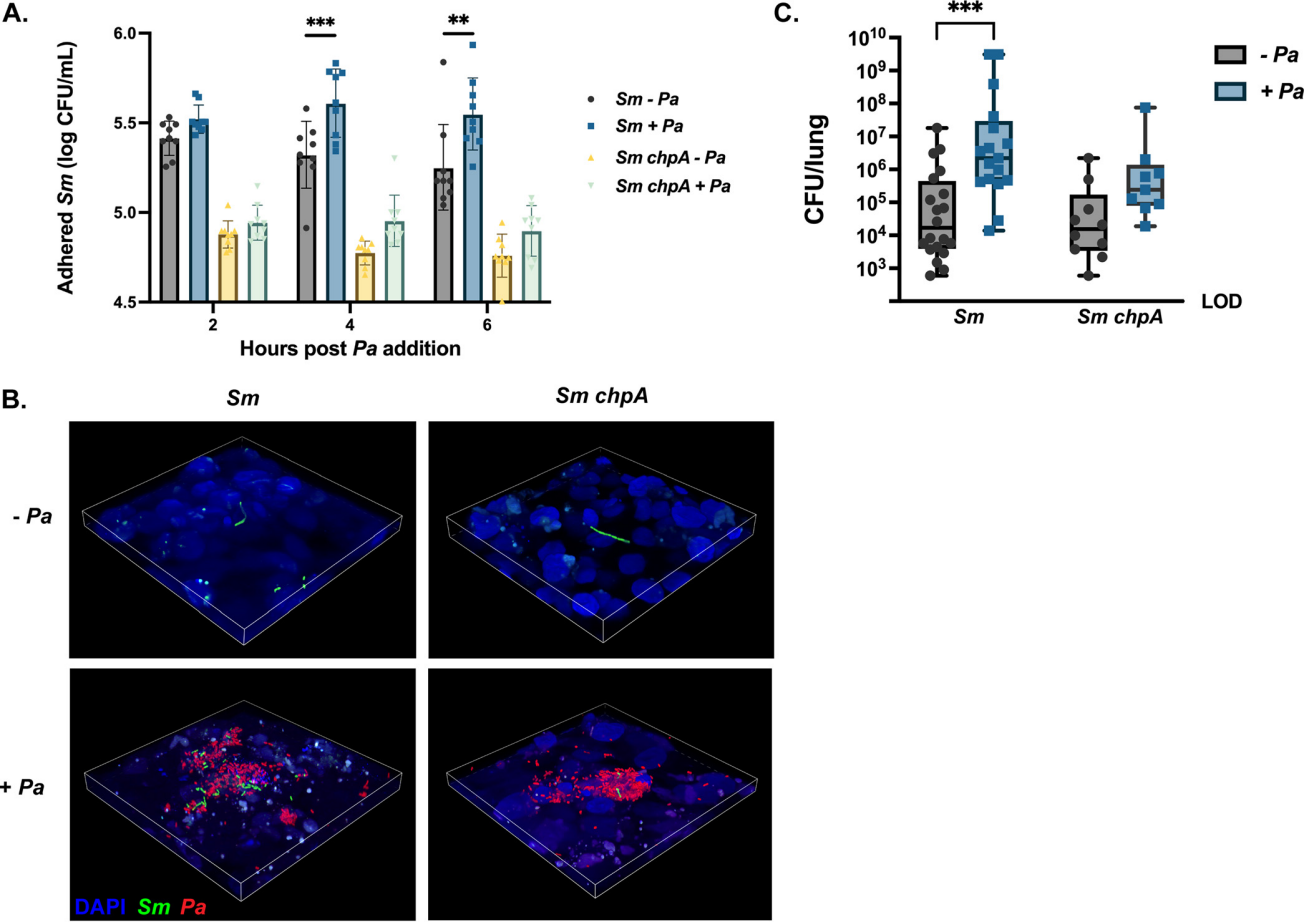
Dysregulation of the type IV pilus abrogates promotion of S. maltophilia adherence. Polarized CFBEs were pretreated with either MEM or ~10^6^ CFU of P. aeruginosa mPA08-31 for 2, 4, or 6 h before the addition of ~10^6^ S. maltophilia K279a or K279a *chpA* for 1 h. (A) Viable colony counts of adherent S. maltophilia (geometric mean ± GSD; *n* = 9 wells). Two-way ANOVA, **, *P* < 0.01; ***, *P* < 0.001). (B) Structural composition of dual-species foci as evaluated via confocal microscopy. Infections were repeated with S. maltophilia K279a (gfp^+^) or K279a *chpA* (gfp^+^) and P. aeruginosa mPA08-31 (mCherry^+^), and CFBEs were visualized with DAPI. Dual-species foci were imaged at ×60 magnification. BALB/cJ mice were intratracheally infected with ~10^8^ CFU of S. maltophilia K279a or K279a *chpA* in the presence and absence of P. aeruginosa mPA08-31 and were euthanized 24 h postinfection. (C) Bacterial burden in the lung enumerated via viable colony counting from lung homogenate (median ± IQR; *n* = 9 to 20; Kruskal-Wallis with Dunn’s multiple comparisons; ***, *P* < 0.001).

The most significantly upregulated type IV pilus-related transcript identified in the RNA-seq experiment, *chpA* (*Smlt3670*), is the histidine kinase subunit of a two-component regulatory system characterized in P. aeruginosa and known to govern twitching motility (34). To see if this gene has an impact on our cooperative phenotype, we created a clean deletion mutant of *chpA* in S. maltophilia K279a. We infected polarized CFBE epithelia with P. aeruginosa mPA08-31 for 2, 4, or 6 h. We then added either S. maltophilia K279a or S. maltophilia K279a *chpA* and quantified adhered bacteria via viable colony counts. As shown previously, prior infection with P. aeruginosa significantly increases the number of adhered S. maltophilia. However, an isogenic S. maltophilia *chpA* mutant strain had significantly decreased adherence to the CFBE epithelial layer, and this was not affected by infection with P. aeruginosa (Fig. 4A). These data were confirmed by CLSM imaging of infected CFBE epithelial cells. The amount of parental S. maltophilia bound to cells increased when P. aeruginosa was present and could be seen binding in the same areas as large clusters of P. aeruginosa. However, the S. maltophilia *chpA* deletion mutant had significantly fewer adherent S. maltophilia, even in areas with abundant P. aeruginosa (Fig. 4B). To confirm that the phenotypes observed *in vitro* were also responsible for the cooperativity we see in the lung, we infected mice with both S. maltophilia and S. maltophilia *chpA* (inoculum, ~10^7^ CFU) in the presence and absence of P. aeruginosa (inoculum, ~10^7^ CFU) for 24 h. We found that coinfection with P. aeruginosa still increased the burden of S. maltophilia K279a *chpA* in the lung compared to single-species infection (Fig. 4C). However, this increase was to a lesser degree than with parental S. maltophilia, with a 289-fold increase in S. maltophilia burden during coinfection compared to a 40-fold increase with S. maltophilia *chpA*.

### Loss of barrier integrity promotes binding of S. maltophilia to the bronchial epithelium.

**FIG 5:**
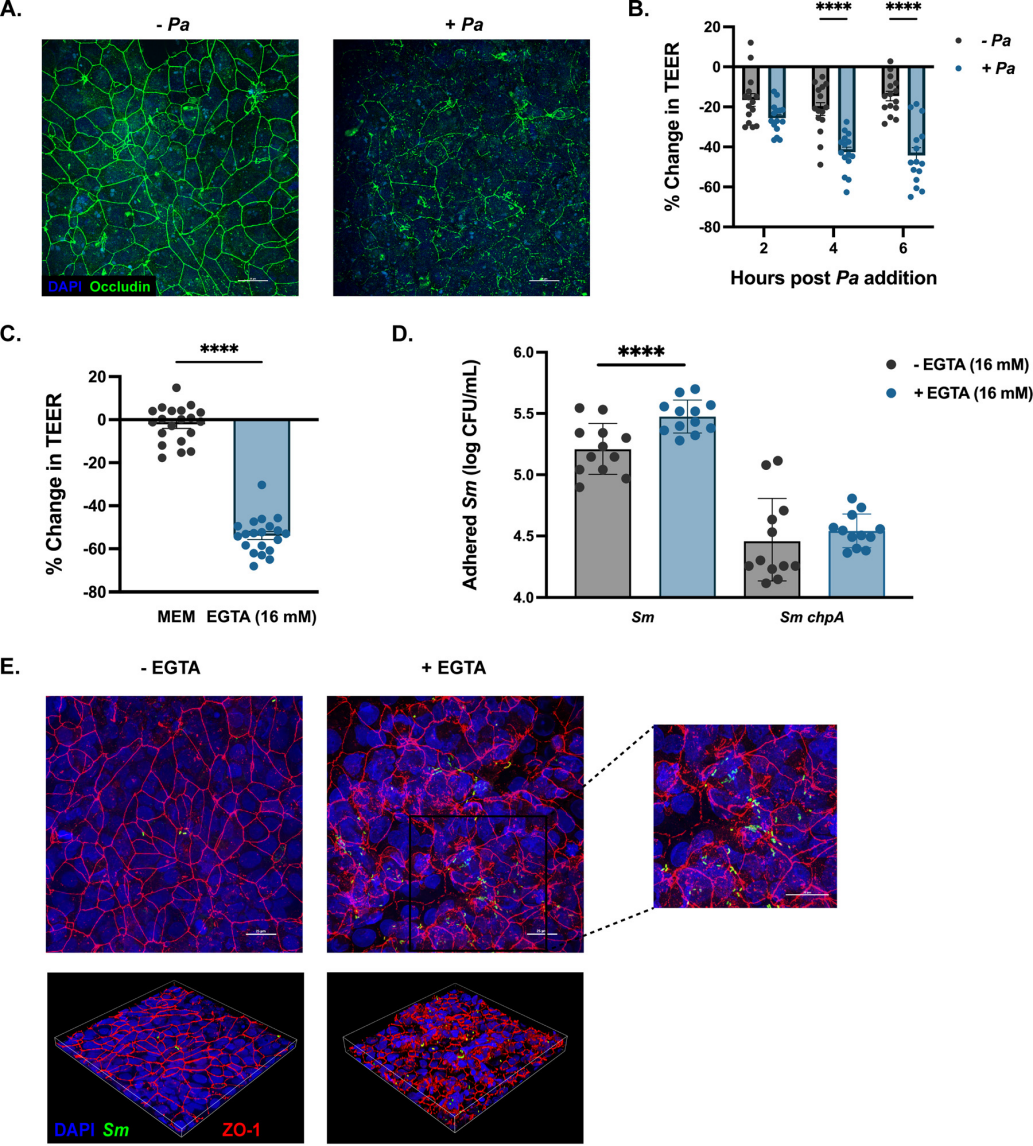
Depolarization of the epithelial membrane is sufficient to promote adherence of S. maltophilia. (A) Confocal imaging of tight junctions stained via occludin from cells treated with either EMEM or ~10^6^ CFU of P. aeruginosa. Cells were imaged at ×60 magnification. (B) Percent change in transepithelial electrical resistance (TEER) across the epithelial membrane after addition of MEM or ~10^6^ CFU of P. aeruginosa (mean ± SEM; *n* = 6 wells; two-way ANOVA; ****, *P* < 0.0001). (C) Percent change in TEER across the epithelial membrane after addition of MEM or 16 mM EGTA for 30 min. (mean ± SEM; *n* = 20 wells; unpaired *t* test, ****, *P* < 0.0001). (D) Viable colony counts of adherent S. maltophilia K279a and K279a *chpA* on CFBEs after a 30-minute pretreatment with either MEM or EGTA (16 mM) (geometric mean ± GSD, *n* = 12 wells; one-way ANOVA; ****, *P* < 0.0001). (E) Confocal imaging of CFBEs pretreated with either MEM (left) or 16 mM EGTA (right) before S. maltophilia inoculation. Cells were stained via ZO-1 (red) for tight junctions and S. maltophilia via anti-*Smlt* rabbit serra (green) and were imaged at ×60 magnification. Inserts were zoomed in 2× for a total magnification of ×120.

P. aeruginosa harbors many virulence factors that affect lung barrier integrity during infection, including several secreted proteases (35, 36). Therefore, we hypothesized that breakdown of tight junctions, and the resulting depolarization of the epithelial cell layer, promotes adherence of S. maltophilia. Infection of polarized CFBEs with P. aeruginosa (MOI = 20) for 6 h dramatically decreased organization of occludin-stained tight junctions compared to cell culture medium alone (Fig. 5A). The transepithelial electrical resistance (TEER), a measurement of monolayer polarity, also decreased significantly over time with the introduction of P. aeruginosa (Fig. 5B). To determine if the increase in S. maltophilia binding could be induced by monolayer depolarization in the absence of P. aeruginosa, we treated cells with 16 mM EGTA, a calcium chelator that has been shown to delocalize tight junction proteins, including occludin and ZO-1 (37, 38). EGTA treatment for 30 min successfully depolarized the epithelial monolayer, with TEER decreasing significantly (*P* < 0.0001) (Fig. 5C). As expected, significantly more S. maltophilia adhered to epithelial cells treated with EGTA than cell culture medium controls (*P* < 0.0001). In contrast, depolarization of the membrane with EGTA did not increase the number of adherent *chpA*-deficient S. maltophilia (Fig. 5D). To determine if S. maltophilia bound to the specific areas of the cell layer with breakdowns in tight junction integrity, we stained for the tight junction protein ZO-1 and S. maltophilia on cell layers with and without EGTA treatment. Without EGTA, tight junctions are intact, and little S. maltophilia is present. After pretreatment with EGTA, tight junction breakdown can be seen, and more S. maltophilia is present (Fig. 5E). These data indicate that breakdown of tight junctions is sufficient to promote colonization with S. maltophilia and that S. maltophilia is likely binding to host factors exposed during breakdown of the epithelial barrier rather than to P. aeruginosa cellular or biofilm components.

### Elastase-mediated damage to the lung epithelium by P. aeruginosa increases S. maltophilia binding.

The production of degradative enzymes by P. aeruginosa is an important virulence factor that can interfere with airway barrier integrity and damage host tissue (35, 36, 39). Of these, elastase B is well characterized for its role in pathogenesis. Elastase B, a secreted protease, is known to break down tight junctions and therefore depolarize epithelial and endothelial cell layers. In combination with toxins secreted by the type III secretion system (T3SS), it also contributes to the ability of P. aeruginosa to invade the epithelium and establish disseminated infection (40).

**FIG 6:**
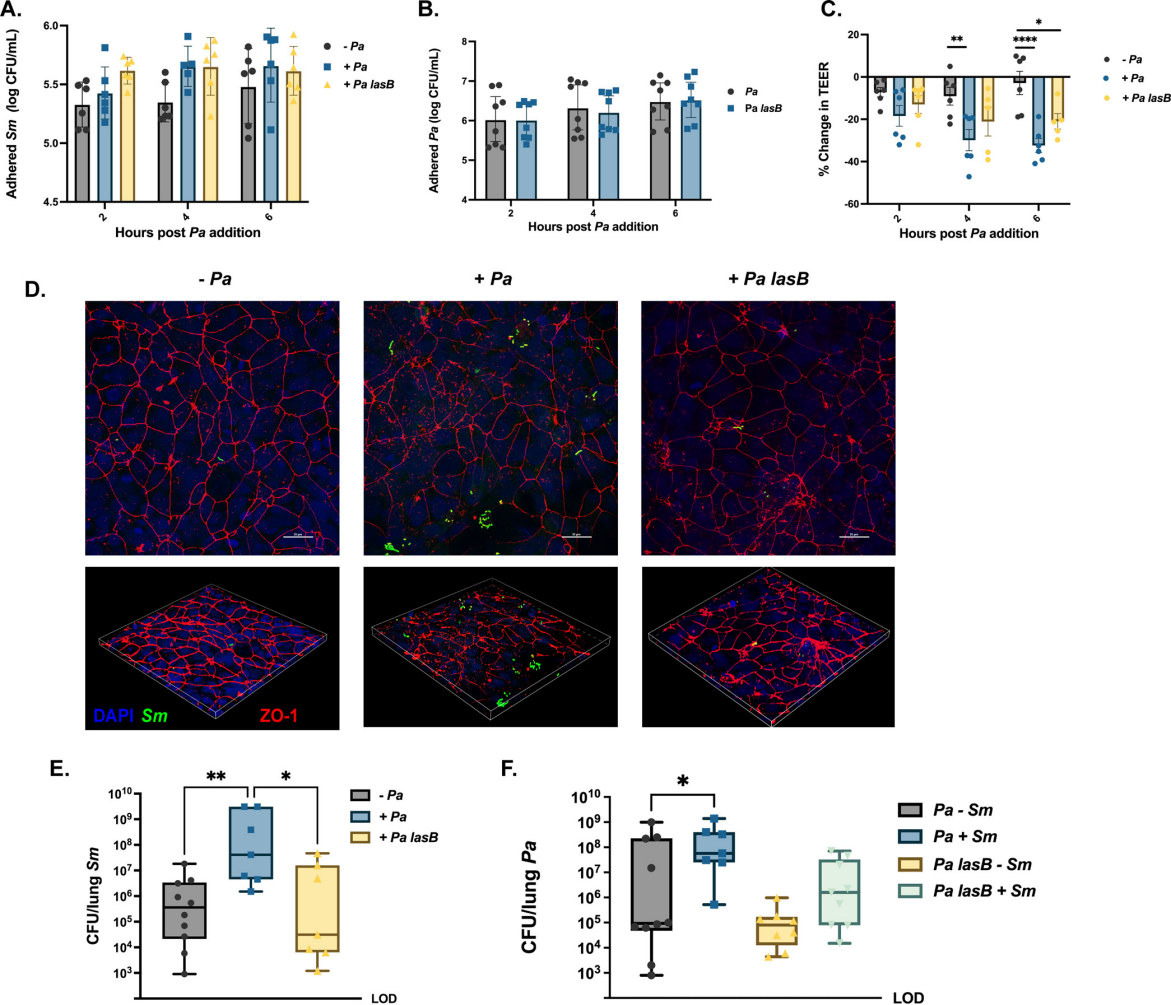
Elastase production by P. aeruginosa contributes to increased persistence of S. maltophilia in the murine lung. Polarized CFBEs were pretreated with MEM, ~10^6^ CFU of P. aeruginosa mPA08-31, or ~10^6^ CFU of P. aeruginosa mPA08-31 *lasB* for 2, 4, or 6 h before the addition of S. maltophilia K279a for 1 h. (A and B) Viable colony counts of adherent S. maltophilia (A) or P. aeruginosa (geometric mean ± GSD; *n* = 8 wells; two-way ANOVA) (B). (C) Percent change in TEER across the epithelial membrane after addition of MEM, mPA08-31, or mPA08-31 *lasB* (mean ± SEM; *n* = 8 wells; two-way ANOVA; *, *P* < 0.05; **, *P* < 0.01; ****, *P* < 0.0001). (D) Confocal imaging of CFBE41s pretreated with either wild type (WT) or elastase-deficient P. aeruginosa before S. maltophilia inoculation. Cells were stained via ZO-1 (red) for tight junctions and S. maltophilia via anti-*Smlt* rabbit sera (green) and were imaged at ×60 magnification. Inserts were zoomed in 2× for a total magnification of ×120. BALB/cJ mice were intratracheally infected with ~10^8^ CFU of S. maltophilia K279a alone or in the presence of P. aeruginosa mPA08-31 or mPA08-31 *lasB* and were euthanized 24 h postinfection. (E and F) Bacterial burden of S. maltophilia (E) and P. aeruginosa (F) in the lung enumerated via viable colony counting from lung homogenate. (median ± IQR; *n* = 7 to 10; Kruskal-Wallis with Dunn’s multiple comparisons; *, *P* < 0.05; **, *P* < 0.01).

Because we found that tight junction degradation was associated with increased adherence of S. maltophilia to host epithelium (Fig. 5), we generated an isogenic P. aeruginosa elastase-deficient mutant (P. aeruginosa mPA08-31 *lasB*), which was used to test the impact of elastase on epithelial integrity and S. maltophilia adherence. We infected polarized CFBE epithelia with P. aeruginosa mPA08-31 or P. aeruginosa mPA08-31 *lasB*, or it was mock infected with cell culture media, for 2 h, 4 h, or 6 h. We then added S. maltophilia K279a, quantified adherent bacteria by viable colony counts, and monitored change in TEER over time. At 4 h and 6 h of incubation, P. aeruginosa promoted S. maltophilia adherence to a greater degree than P. aeruginosa *lasB* (Fig. 6) despite no difference in P. aeruginosa burden between parental and knockout strains (Fig. 6A and B). However, this did not reach statistical significance at any time point tested. Consistent with these results, P. aeruginosa decreased TEER across the epithelial monolayer to a greater degree than P. aeruginosa *lasB*, although this was again not statistically significant (Fig. 6C). These results were confirmed via confocal imaging of cell monolayers stained for S. maltophilia and tight junctions (ZO-1). More S. maltophilia was present when cells were preinfected with P. aeruginosa mPA08-31 than when infected with mPA0831 *lasB*. ZO-1 organization was also slightly better preserved in the cell layer infected with the *lasB* mutant than in the parent strain (Fig. 6D). To evaluate the contribution of elastase to our *in vivo* phenotype, we repeated mouse infection experiments using P. aeruginosa and P. aeruginosa *lasB* in conjunction with S. maltophilia. We found that coinfection with P. aeruginosa again significantly increased the S. maltophilia bacterial burden but that P. aeruginosa *lasB* did not increase the burden of S. maltophilia above that of single-species infection (Fig. 6E). P. aeruginosa *lasB* resulted in a 3-fold increase in S. maltophilia burden during coinfection compared to a 350-fold increase with the parent strain. However, loss of LasB decreased persistence of P. aeruginosa in single-species infection, though not to a statistically significant degree, making interpretation of changes in S. maltophilia burden during dual-species infection difficult. These results indicate that elastase production by P. aeruginosa, and likely the resulting inflammation and lung damage, may be factors for the cooperative behavior of these two organisms in the murine lung.

## DISCUSSION

Respiratory infections are a substantial contributor to morbidity and mortality in pulmonary diseases. While many pathogens responsible for these infections have been identified, the advent of culture-independent detection methods has led to an appreciation of the complex ecology of the lung and the impact that interspecies interactions have on patient outcomes. Our prior work showed that colonization and persistence of S. maltophilia in the murine lung was significantly increased in dual-species infections with P. aeruginosa. We also demonstrated that this increased persistence resulted in a higher mortality rate among the infected mice (24). In the present study, we showed that epithelial barrier damage by P. aeruginosa mediates increased binding and persistence of S. maltophilia in the murine lung.

Previous metrics of virulence indicated that P. aeruginosa infection drives the host response during dual-species infection and that the response to S. maltophilia is comparatively less severe (24). Our results from RNA sequencing experiments are concordant with this finding, with the majority of genes upregulated during dual-species infection also upregulated during P. aeruginosa infection compared to S. maltophilia alone. Only 21 total genes were differentially regulated between all three conditions, and pathway analysis was unable to identify specific pathways associated with these. Principal-component analysis of host gene expression suggests that infection with P. aeruginosa elicited a similar host response to concurrent infection with both bacterial species. Compared to S. maltophilia infection, host genes involved in both cytokine production and cytokine-mediated signaling pathways were upregulated in dual species-infected mice and mice infected with P. aeruginosa alone. However, these differences might be due in part to reads coming from recruited cells, as previous studies found a dramatic neutrophil influx as a result of infection with P. aeruginosa or during dual-species infections (24). Although these two organisms share many cellular structures able to elicit a strong immune response (endotoxin, flagella, pili, etc.), the ability of P. aeruginosa to damage the lung epithelium through the release of toxins is likely contributing to this response. Genes involved in the cell-to-cell adhesion pathway were also upregulated, indicating a breakdown of barrier integrity.

Host-microbe RNA-seq is a powerful technique that allows for a snapshot of gene expression from both the pathogen and the host in different infection contexts. An important limitation of this technique is the ability to obtain adequate bacterial reads from RNA samples that are overwhelmingly made up of host transcripts. For example, we found that a sequencing depth of ~30 million reads from lung samples of mice infected with S. maltophilia alone yielded on average 0.01% of reads mapping to S. maltophilia. To overcome this limitation, we employed a recently published selective mRNA capture technique known as PatH-Cap (pathogen-hybrid capture), which allows for pathogen-specific coding sequences to be enriched from a pool of host and noncoding transcripts (28). For the previously mentioned example that contained 0.01% bacterial reads, application of PatH-Cap increased bacterial reads to 6.97%, a 697-fold increase in relative abundance. PatH-Cap was even more effective with RNA samples from dual species-infected mice. The initial bacterial read percentage was 10-fold higher at 0.10%, and the final read percentage was at ~80%, a 770-fold increase in relative abundance. This illustrates that PatH-Cap is highly dependent on the percentage of input bacterial RNA in the pooled RNA sample. Given sufficient starting material, we have demonstrated that PatH-Cap is a renewable and cost-effective strategy for investigating bacterial RNA expression in disease-relevant contexts, overcoming a major limitation in the bacterial pathogenesis field.

The *in vivo* bacterial RNA-seq results indicated that genes involved in control and biogenesis of the type IV pilus are upregulated in the context of dual-species infection in the lung. This included *chpA*, the histidine kinase subunit of a two-component system that, although largely uncharacterized in S. maltophilia, is known to regulate twitching motility, mechano- and chemotaxis, and cAMP regulation in P. aeruginosa (29, 34, 41). Deletion of *chpA* in S. maltophilia dramatically decreased adherence to CFBEs. Interestingly, previous reports indicate that both flagella and fimbriae of S. maltophilia can mediate binding to epithelial cells (33, 42, 43). However, this work was limited to abiotic surfaces, cell lines not derived from pulmonary epithelium, and nonpolarized epithelial layers. Extensive work in P. aeruginosa has shown that both flagella and the type IV pilus can mediate binding to epithelial cells but showed vastly different substrate specificity, with the type IV pilus binding preferentially to host *N*-glycans on the apical side of a polarized epithelium and flagella binding preferentially to heparin sulfate proteoglycans on the basal surface (44). It therefore seems likely that S. maltophilia, like P. aeruginosa, may use several different adherence mechanisms in a context-dependent or redundant manner.

While we know that S. maltophilia and P. aeruginosa can be isolated together out of the lung, this is certainly not the only risk factor for acquisition of S. maltophilia. Decreased lung function, previous antibiotic use, and an indwelling device all predispose to S. maltophilia infection (45). The results here, although important for understanding a multispecies interaction, also clarify how a damaged lung environment might be sufficient to promote S. maltophilia binding, as would be the case in people with late-stage CF disease. EGTA experiments demonstrate that depolarization of the cell monolayer in the absence of P. aeruginosa is sufficient to induce increased S. maltophilia binding. Damage and subsequent depolarization of epithelial membranes can expose receptors or ligands that allow for more effective pathogen binding, and the role of previous lung damage in establishment of future infection is well characterized for many other pathogens (46, 47). The mechanism described here is likely not unique to these two organisms but may represent a larger paradigm for dual-species cooperativity in the human lung. Several alternative mechanisms might also be at play, including changes in cellular immune response or nutrient availability. An important next step in this work would be the identification of the host factor responsible for S. maltophilia binding, particularly in the context of a damaged lung environment (48).

With the advent of effective modulator therapies in CF, the rapid decline in lung function and aggressive cycle of inflammation associated with chronic infection with P. aeruginosa and other CF pathogens may become a thing of the past. Our data suggest that this might bring with it a subsequent decrease in susceptibility to S. maltophilia in this patient population. However, the widespread use of ventilators in response to patients with severe COVID-19 infection has brought its own wave of VAP cases, many of which involve infection with S. maltophilia and are polymicrobial. With the highly antibiotic-resistant nature of S. maltophilia, new antimicrobial strategies are needed. Characterization of adherence machinery of S. maltophilia and the specific host factors that allow for successful infection could be a first step in successful control of this important airway opportunist.

## MATERIALS AND METHODS

### Strains and growth conditions.

S. maltophilia K279a is a widely used model strain with a fully annotated genome sequence, originally isolated from a patient with bacteremia in the United Kingdom (15); this strain and the S. maltophilia K279a-green fluorescent protein (GFP) derivative were provided by M. Herman (Kansas State University). P. aeruginosa mPA08-31 was originally isolated from the sputum of a patient with CF and was provided by S. Birket (University of Alabama at Birmingham). P. aeruginosa mPA08-31-mCherry+ was constructed by transforming parent strains with plasmid pUCP19+mCherry provided by D. Wozniak (Ohio State University). All strains were routinely cultured on Luria-Bertani (LB) agar (Difco) or in LB broth. S. maltophilia strains were streaked for colony isolation before inoculating into LB broth and shaking overnight at 30°C and 200 rpm. P. aeruginosa strains were streaked for colony isolation before inoculating into LB broth and shaking overnight at 37°C and 200 rpm.

### Mouse respiratory infections.

BALB/cJ mice (8 to 10 weeks old) were obtained from Jackson Laboratories (Bar Harbor, ME). Mice were anesthetized with isoflurane and intratracheally infected with either S. maltophilia, P. aeruginosa, or both (~10^7^ CFU each in 100 μL phosphate-buffered saline [PBS]). Mice were euthanized 24 h postinfection, and the left lung of each mouse was harvested and homogenized in 500 μL of sterile PBS for viable plate counting. The homogenates from single-species infections were serially diluted in PBS and plated on LB to obtain viable CFU counts. The homogenates from dual-species infections were plated on M9 minimal medium (49) to enumerate P. aeruginosa and LB agar containing gentamicin (50 μg/mL) to enumerate S. maltophilia. All samples from dual-species infections were also plated on LB for total bacterial counts. All mouse infection protocols were approved by the UAB Institutional Animal Care and Use Committees.

### RNA library preparation.

For RNA isolation from the lung, BALB/cJ mice (8 to 10 weeks old) were obtained from Jackson Laboratories (Bar Harbor, ME). Mice were anesthetized with isoflurane and intratracheally infected with either S. maltophilia, P. aeruginosa, or both (~10^7^ CFU each in 100 μL PBS). Mice were euthanized 24 h postinfection, and lungs were inflated with RNAlater to preserve RNA integrity. Whole lungs were homogenized, and cells were lysed by bead beating (0.1 mm silica) in TRIzol reagent (Invitrogen), and a full-lung RNA extraction was performed using a standard protocol (50). Extracted RNA samples were sent to Genewiz (South Plainfield, NJ) for DNase treatment, host and bacterial rRNA depletion (Ribo-Zero Gold rRNA removal kit [Epidemiology]; Illumina), and library preparation using standard protocols. For bacterial RNA-seq, single-end directional samples were DNase treated (DNase I; NEB) using a standard protocol before being run through a second TRIzol extraction to purify the sample of enzyme. Clean RNA was first rRNA depleted using the NEBNext rRNA depletion kit (human/mouse/rat) (NEB) before being prepared for sequencing using the NEBNext Ultra II Directional RNA library prep kit (NEB) and tagged for multiplexing via the NEBNext multiplex oligos for Illumina (NEB).

### Pathogen-hybrid capture.

A pathogen-specific probe list for S. maltophilia was generated using previously published methods (28). Briefly, 100-bp probes were generated to cover annotated coding sequences of S. maltophilia, with 15-bp spacer sequences added on either end to allow for amplification of the probe library as follows: 5′ SPACER, ATCGCACCAGCGTGT, and 3′ SPACER, CACTGCGGCTCCTCA.

Probes completely covered the sense strand of each gene and were also generated to tile every other 100 bp of the antisense strand for a total of 68,704 probe sequences (see Fig. S1 in the supplemental material). Probes with significant homology to the mouse genome or bacterial noncoding RNAs (*P* < 0.05 with BLAST analysis) were manually removed from the list. The resulting oligonucleotide pool was synthesized by GenScript (Piscataway, NJ). Before hybridization, DNA oligonucleotides were amplified and then transcribed (MEGAshortscript T7 transcription kit; Ambion), with added biotin-16-UTP (Roche) to generate biotinylated RNA probes.

Prepared cDNA libraries were hybridized to the generated pathogen-specific RNA probes using previously described methods (28) with a few modifications. Briefly, the synthesized probes and the prepared cDNA library were incubated in hybridization buffer for 24 h at 68°C. The following blocking primers were modified for compatibility with NEB multiplexing primers, and a 3′ ddc′ modification was added to maintain barcoding: hybridization primer, forward, AATGATACGGCGACCACCGAGATCTACACTCTTTCCCTACACGACGCTCTTCCGATCT/3ddC/, and reverse, CAAGCAGAAGACGGCATACGAGATNNNNNNNNGTGACTGGAGTTCAGACGTGTGCTCTTCCGATCT/3ddC/.

Mouse cot-1 DNA was also swapped for human cot-1 DNA in the hybridization buffer to account for host differences. Hybridized cDNA was isolated via streptavidin beads and then eluted. A diagnostic quantitative PCR (qPCR; Kapa library quantification kit, Roche) was used to determine the appropriate number of amplification cycles, and enriched samples were amplified using universal primers that maintained sample barcoding before sequencing. All primers used in these experiments are detailed in Table S3.

### Sequencing, alignment, and analysis.

For host RNA-seq, paired-end strand-specific RNA sequencing was performed using an Illumina HiSeq2 with ~25,000,000 reads per sample. Reads were trimmed with TrimGalore! (v.0.4.4) and Cutadapt (v.1.9.1) and evaluated for quality with FastQC. Reads were aligned to the mouse transcriptome generated from Ensembl gene annotations (build GRCm39/mm39) using the STAR aligner (v. 2.7.3a). Read counts were obtained via Subread FeatureCounts, and differential expression analysis was performed with DESeq2, with a *P*-value cutoff of <0.01. Pathway analysis was performed using clusterProfiler (v.4.4.1) with Gene Ontology (biological processes) and KEGG pathway databases. Single-end strand-specific sequencing was performed on samples enriched for pathogen-specific RNA via hybridization (~30,000,000 reads/sample) via Illumina NextSeq500. These reads were again trimmed with TrimGalore! (v.0.4.4) and Cutadapt (v.1.9.1) and evaluated for quality with FastQC. Reads were aligned to the published genome of S. maltophilia K279a using the STAR aligner (v.2.7.3a). Read counts were obtained via Subread FeatureCounts, and differential expression analysis was performed with DESeq2. Final analysis of significantly up- and downregulated genes from S. maltophilia in the context of dual-species infection was restricted to those genes with transcripts detected in at least 2/4 samples from each group.

### Cell culture.

Cystic fibrosis bronchial epithelial cells (CFBE41o-) cells, henceforth referred to as CFBEs, are an immortalized human bronchial epithelial cell line homologous for the F508del mutation in CFTR (51). Cells were routinely cultured in minimal essential medium (MEM; Corning) with 10% fetal bovine serum (FBS) and were polarized by seeding at a density of 5 × 10^6^ on the apical surface of transwells (0.4 μm; Corning) and growing at 37°C for 7 days before removing the apical media and growing for an additional 7 days at the air-liquid interface. Polarization of the epithelial membranes was confirmed via transepithelial electrical resistance measurements performed via EVOM2 volt/ohm meter (World Precision Instruments).

### Adherence assays.

To measure the adherence of S. maltophilia to CFBEs after prior infection with P. aeruginosa, cells were inoculated with ~10^6^ CFU (MOI = 20) of P. aeruginosa mPA08-31 in MEM (no FBS). The media on the basal side of the chamber was also replaced with FBS-free medium before incubation. Bacteria were incubated on the cells for 2, 4, or 6 h before the supernatant was removed from the apical chamber. Cells were then inoculated with ~10^6^ CFU (MOI = 20) of S. maltophilia K279a and incubated for an hour. Cells were washed twice with sterile 1× PBS before being scraped from the Transwell membrane, diluted, and plated on differential medium to enumerate the bacterial burden. TEER was measured for each well both before infection and at the end of P. aeruginosa mPA-0831 infection at each time point specified via EVOM2 volt/ohm meter. Changes in epithelial resistance were expressed as the percent change from TEER measurement at baseline.

For EGTA exposure experiments, cells were treated apically with plain MEM or MEM with 16 mM EGTA for 30 min at 37°C. The medium was then removed, and cells were inoculated with ~2 × 10^6^ CFU (MOI = 20) of S. maltophilia and incubated for an hour. Bacteria were enumerated as previously described.

### Immunofluorescence staining and confocal microscopy.

For imaging of dual-species infections, cell layers were infected as described above with P. aeruginosa mPA08-31 (mCherry^+^) and S. maltophilia K279a (gfp^+^) or K279a *chpA* (gfp^+^). Cells were fixed with 4% paraformaldehyde overnight at 4°C. Cells were then rehydrated with 1× PBS and stained with DAPI (4′,6-diamidino-2-phenylindole). Filters were mounted with ProLong Diamond antifade (Invitrogen) and were imaged using z-stacks via confocal laser scanning microscopy (CLSM) using a 60× objective.

For imaging of tight junctions, cells were stained via immunofluorescence. Cells were fixed as previously described, with a few modifications (52). In brief, cells were fixed in 1:1 acetone methanol at 20°C for 10 min before rehydrating in 1× Tris-buffered saline (TBS). Cell layers were blocked with 1× TBS with 3% bovine serum albumin (BSA) for 30 min before staining. Cells were incubated with primary antibody (rabbit polyclonal anti-occludin or donkey anti-goat ZO-1 for tight junctions and an anti-*Smlt* rabbit sera cross adsorbed against P. aeruginosa for staining of S. maltophilia) for 1 h at room temperature. Filters were then washed and incubated with secondary antibody for 1 h at room temperature before being stained with DAPI and mounted with ProLong Diamond antifade. Filters were again imaged using z-stacks via CLSM using a 60× objective. For insets, images were zoomed in 2× for a total magnification of ×120.

CLSM was performed using a Nikon-A1R HD25 confocal laser microscope (Nikon, Tokyo, Japan). Images were acquired and processed using the NIS-Elements 5.0 software.

### Bacterial deletion mutants.

An unmarked clean deletion of Smlt3670 (*chpA*) in S. maltophilia was produced via two-step homologous recombination as has been previously described for P. aeruginosa (53). DNA fragments of 500 to 1,000 bp upstream and downstream of each gene were inserted in pEX18Tc using the Gibson assembly cloning kit (NEB) using standard protocols from the manufacturer. Plasmids were transformed into Escherichia coli DH5α before introduction into S. maltophilia K279a via triparental conjugation with helper strain PRK2013 as previously described (54). Clean deletion was confirmed by PCR amplification of the designated region. The unmarked deletion mutant of *lasB* in P. aeruginosa mPA08-31 was produced using the methods detailed above, but with a pEX18Gm backbone. For each mutant in S. maltophilia or P. aeruginosa, at least two independently derived mutants were evaluated, and whole-genome sequencing was performed showing a lack of secondary mutations. RNA sequencing of parent and *chpA* mutant S. maltophilia showed a lack of differential expression in genes downstream of *chpA* (Fig. S4).

### Statistical analyses.

Unless otherwise noted, graphs represent sample means ± standard error of the mean (SEM). For nonparametric analyses, differences between groups were analyzed by Kruskal-Wallis test with the uncorrected Dunn’s test for multiple comparisons. For normally distributed data sets (as determined by the Shapiro-Wilk normality test), a one-way analysis of variance (ANOVA) was used with Tukey’s multiple-comparison test. For analyses with more than one factor, a two-way ANOVA was used. All statistical tests were performed using GraphPad Prism 9 (San Diego, CA).

### Data availability.

Sequencing data generated for all samples included in this study are deposited in the NCBI Sequence Read Archive. All RNA-seq data files are publicly available via NCBI GEO GSE216847. Accession numbers for individual sample sequencing read libraries are provided in the supplemental information.
