# Supplementary material for: Pseudomonas aeruginosa Promotes Persistence of Stenotrophomonas maltophilia via Increased Adherence to Depolarized Respiratory Epithelium
Source: Microbiol Spectr. 2022 Dec 6;11(1):e03846-22. doi: 10.1128/spectrum.03846-22 (PMC9927254; doi:10.1128/spectrum.03846-22)
Supplement: Supplemental file 1 — Supplemental material. Download spectrum.03846-22-s0001.pdf, PDF file, 1.3 MB [file spectrum.03846-22-s0001.pdf]

RNA-seq round 1					
**Sample**	**Group**	**Input reads**	**Mouse % mapped**	**K279a reads mapped**	**K279a % mapped**
m22-1	K279a	22265427	73.69	253	0
m22-2	K279a	22493652	77.98	369	0
m22-3	K279a	23983260	75.88	671	0
m22-4	K279a	29492187	77.24	935	0
m22-5	K279a	19788525	79.33	1217	0.01
m22-6	Dual	24141258	72.4	636187	2.64
m22-7	Dual	25028937	77.2	1513	0.01
m22-8	Dual	28801422	78.34	1543	0.01
m22-9	Dual	23450730	77.34	649	0
m22-10	Dual	28705764	80	459	0
m22-11	mPA0831	20757985	77.23	156	0
m22-12	mPA0831	22922506	80.44	428	0
m22-13	mPA0831	27687141	77.49	358	0
m22-14	mPA0831	28341461	78.39	241	0
m22-15	mPA0831	24855211	80.38	158	0

RNAseq round 2 (PatH-Cap)					
**Sample**	**Group**	**Input reads**	**Mouse % mapped**	**K279a reads mapped**	**K279a % mapped**
m25-3K	K279a	28024276	47.54	9661463	34.48
m25-19K	K279a	31074448	55.56	4507	0.01
m25-20K	K279a	34731646	53.56	20376	0.06
m25-21K	K279a	25135729	52.95	563941	2.24
m25-23K	Dual	37830227	4.41	30333084	80.18
m25-24K	Dual	40904016	4.97	33113005	80.95
m25-25K	Dual	34263984	4.52	29489707	86.07
m25-26K	Dual	33974557	3.83	29973459	88.22

Table S1. RNA sequencing mapping statistics. Reads from sequencing were trimmed then aligned to *Mus musculus* (mm10) and *S. maltophilia* K279a genomes. Percent of total reads mapped to each genome are reported. Round 1 of sequencing was performed on whole lung RNA extractions, with depletion for rRNA from host and bacteria. Round 2 of sequencing was performed on whole lung RNA, after rRNA depletion and pathogen-specific enrichment for *S. maltophilia* K279a.

Gene ID	L2FC	*P* value	*P* adj.	Gene
Smlt3668	4.2915	1.08E-03	9.04E-03	chpC
Smlt3669	4.5002	1.20E-04	1.79E-03	chpB
Smlt3670	4.5334	5.04E-07	4.70E-05	chpA
Smlt3671	1.9556	2.08E-01	3.81E-01	pilJ
Smlt3672	6.0615	2.50E-06	1.17E-04	pilI
Smlt3673	2.8634	3.72E-02	1.21E-01	pilH
Smlt3674	6.1035	1.39E-04	1.97E-03	pilG
Smlt3756	0.9631	4.66E-01	6.47E-01	pilB
Smlt3757	6.4439	5.55E-07	4.83E-05	pilA
Smlt3758	-0.1245	9.38E-01	9.69E-01	pilA
Smlt3759	1.4679	2.81E-01	4.69E-01	pilC
Smlt3821	8.4000	1.23E-11	2.92E-08	pilQ
Smlt3822	5.6456	4.88E-04	4.98E-03	pilP
Smlt3823	6.5925	7.51E-06	2.63E-04	pilO
Smlt3824	4.0862	5.39E-04	5.34E-03	pilN
Smlt3825	3.4516	1.18E-02	5.21E-02	pilM
Smlt1089	4.9151	2.41E-03	1.63E-02	pilT
Smlt1090	2.9692	1.20E-02	5.26E-02	pilU
Smlt0612	4.8630	1.11E-03	9.14E-03	pilU

Table S2. *S. maltophilia* K279a gene expression of type 4 pilus (T4P)-associated pathways. Differential expression analysis of pilus related genes during single or dual species infection. Log fold changes indicate expression differences in dual species infections relative to single species infections. Green indicates p-values less than 0.05.

Flagellar regulation and biosynthesis operons

	GeneID	baseMean	log2FoldChan	lfcSE	stat	pvalue	padj
flgN	Smlt2321	9.51830149	1.53306512	1.93614882	0.79181161	0.42847053	NA
flgM	Smlt2320	9.69965616	1.4108005	2.56368037	0.55030281	0.5821117	NA
flgA	Smlt2319	2.53099176	-0.6004712	3.34057397	-0.1797509	0.85734812	NA
cheV	Smlt2318	9.38210884	1.29530771	3.24957641	0.39860817	NA	NA
flgB	Smlt2317	57.2199726	-0.8728489	1.46324604	-0.5965155	NA	NA
flgC	Smlt2316	49.8155532	-1.4212801	2.30748591	-0.6159431	0.53793206	0.70870603
flgD	Smlt2315	22.3450397	2.91216342	1.64717987	1.76796929	0.07706604	0.2024439
flgE	Smlt2314	27.9556551	3.29584789	1.56586815	2.1048055	0.03530824	0.11703465
flgF	Smlt2313	26.9287775	1.32970874	2.3369055	0.56900407	0.56935338	0.73063454
flgG	Smlt2312	19.6207821	2.45956674	2.36830712	1.03853369	0.29902166	0.48695727
flgH	Smlt2311	18.7396689	2.64906301	1.66099689	1.59486332	0.11074281	0.25577694
flgI	Smlt2310	15.4240354	2.30486955	1.76819946	1.30351219	0.19239996	0.36174955
flgJ	Smlt2309	11.739749	1.70194313	2.46256716	0.69112557	0.48948663	NA
flgK	Smlt2308	75.6626135	0.30294396	1.5431173	0.19631946	0.84436013	0.9200325
flgL	Smlt2307	23.8305992	1.39408921	1.87603268	0.74310497	0.45741813	0.63926314
fliC1	Smlt2306	54.8398393	3.02026489	1.71517755	1.7609051	0.07825447	0.20417396
fliC2	Smlt2305	295.925615	3.43797172	1.37440536	2.50142485	0.01236947	0.05358942
fliC3	Smlt2304	390.305996	2.9992021	1.23824915	2.42213137	0.01542977	0.06353678
fliD	Smlt2303	93.3736099	0.30205465	1.41583236	0.21334069	0.83106124	0.91375095
fliS	Smlt2302	7.7654045	-0.8797493	3.0297913	-0.2903663	NA	NA
fliE	Smlt2290	6.07674231	0.70764406	2.79145463	0.25350369	0.79987901	NA
fliF	Smlt2289	9.29875952	1.31555137	2.84687689	0.46210336	0.6440072	NA
fliG	Smlt2288	24.3158478	3.07826128	1.58641122	1.94039303	0.05233194	0.15423641
fliH	Smlt2287	6.61899967	0.78147339	3.09022459	0.25288563	0.80035659	NA
fliI	Smlt2286	31.4380276	0.4611068	2.17029383	0.21246284	0.83174596	0.91375095
fliJ	Smlt2285	10.6667403	-2.7214013	2.86796256	-0.9488971	0.34267297	NA
fliK	Smlt2284	25.492518	-0.5427848	2.24213616	-0.2420838	0.80871526	0.90081074
fliL	Smlt2283	4.77471903	0.32179641	2.6801256	0.12006766	0.90442955	NA
fliM	Smlt2282	14.4390225	2.09285135	2.05653688	1.01765807	0.30884048	0.49763924
fliN	Smlt2281	1.31771528	-1.5420759	3.34125105	-0.4615265	0.64442091	NA
fliO	Smlt2280	1.57669899	-1.2832259	3.34101907	-0.3840822	0.70091753	NA
fliP	Smlt2279	8.68844208	1.30870501	2.25060492	0.58149034	0.56091003	NA
fliQ	Smlt2278	7.0510967	0.92253659	2.78768648	0.33093269	0.74069533	NA
fliR	Smlt2277	8.12392154	1.23598141	2.15165557	0.57443274	0.565675	NA
flhB	Smlt2274	5.34671034	0.56033314	2.50595971	0.22360022	0.82306839	NA
flhA	Smlt2273	11.6133751	1.84674429	1.87036252	0.98737238	0.32346012	NA
flhF	Smlt2272	12.2836941	-1.3929103	2.47132709	-0.5636285	NA	NA
fleN	Smlt2271	7.69922978	1.06152848	2.68451545	0.39542647	0.69252818	NA
fliA	Smlt2270	5.55444883	0.5588852	2.9874472	0.18707785	0.8515996	NA
cheY	Smlt2269	5.77957443	0.68103957	2.45345928	0.2775834	0.78133218	NA
cheZ	Smlt2268	4.04089803	0.07439501	3.34029916	0.02227196	0.98223102	NA
cheA	Smlt2267	38.5066069	2.2303121	1.81909124	1.2260584	0.2201767	0.39606054
motC	Smlt2266	18.183116	2.35671477	2.33282074	1.01024255	0.3123791	0.50056221
motD	Smlt2265	39.0577151	2.26707475	1.79983298	1.25960285	0.20781267	0.38004267
rpoN	Smlt2297	40.5962904	2.49625083	1.74583954	1.42982833	0.1527663	0.31815107
	Smlt2296	36.1359539	3.69039966	1.53706549	2.40093846	0.01635309	0.06653716
fleQ	Smlt2295	114.078013	4.90860947	1.39253616	3.52494219	0.00042358	0.00450837

Fimbrial genes

	GeneID	baseMean	log2FoldChan	lfcSE	stat	pvalue	padj
smf-1	Smlt4180	83.905904	-1.908006	1.70854115	-1.1167457	0.26410307	0.44895644

Table S3. *S. maltophilia* K279a gene expression of flagellar and fimbrial pathways. Differential expression analysis of flagellar and fimbrial genes during single or dual species infection. Log fold changes indicate expression differences in dual species infections relative to single species infections. Green indicates p-values less than 0.05.

	Primer Name	Primer Sequence 5' - 3'
**Amplification of *Smlt3670* (*chpA*)**	Smlt3670 FWD	TCG GAA ATC GTG CTG GAA G
	Smlt3670 REV	GTG AAA CCA GAG GCG AAG AT
**Plasmid contruction for clean deletion of *Smlt3670* (*chpA*)**	Smlt3670_upstream_FWD	ATC CCC GGG TAC CGA GCT CGG GTG CGC CGG TGG CGC ATA CAG G
	Smlt3670_upstream_REV	CCG GCG CAC CAG CCC AGG CGG CCC CGG C
	Smlt3670_downstream_FWD	CGC CTG GGC TGG TGC GCC GGT GGC GCA TAC AGG
	Smlt3670_downstream_REV	CAG CTA TGA CCA TGA TTA CGA GCC CAG GCG GCC CCG GC
**Amplification of *lasB***	lasB FWD	ACTCGATGAAACGGGTGATG
	lasB REV	CCTTCTACCCGAAGGACTGATA
**Plasmid contruction for clean deletion of *lasB***	lasB_upstream_FWD	ATC CCC GGG TAC CGA GCT CGG TCC TAC CGG GAT TTC CGC ATT CGC CG
	lasB_upstream_REV	ACT GAA CAA GGC TCG GTG GCC CCG GCC G
	lasB_downstream_FWD	GCC ACC GAG CCT TGT TCA GTT CTC CTG GTT TTT TCA GGC C
	lasB_downstream_REV	CAG CTA TGA CCA TGA TTA CGG AGG CGC TGG CGC AGC TC
**Amplification of cDNA library (maintains barcoding) - PatH-Cap**	NEB hyb primer FWD	AAT GAT ACG GCG ACC ACC GAG ATC TAC ACT CTT TCC CTA CAC GAC GCT CTT CCG ATC T/3ddC/
	NEB hyb primer REV	CAA GCA GAA GAC GGC ATA CGA GAT NNN NNN NNG TGA CTG GAG TTC AGA CGT GTG CTC TTC CGA TCT /3ddC/
**Amplification of cDNA library (no barcoding) - PatH-Cap**	P5 primer	AAT GAT ACG GCG ACC ACC GAG ATC TAC ACT CTT TCC CTA CAC GAC GCT CTT CCG ATC
	P7 primer	CAA GCA GAA GAC GGC ATA CGA GAT

Table S4. Primers used in present study. *lasB* and *chpA* primers were used for the construction of plasmids for clean deletion via homologous recombination.

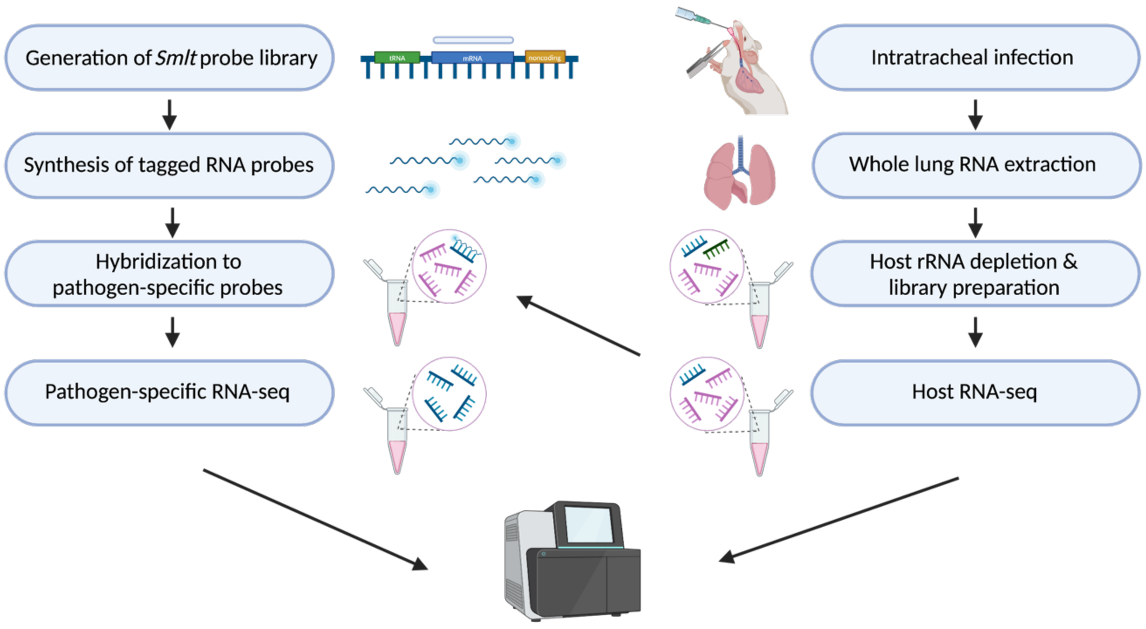

Figure S1. RNA isolation and sequencing methodology

A schematic depicting the workflow of host-pathogen RNA seq using pathogen-hybrid capture (PatH-cap). BALB/cJ mice were intratracheally infected with ~10^7 CFU of *S. maltophilia* K279a and *P. aeruginosa* mPA08-31 alone, and in combination, and groups were euthanized at 24 hours post-infection. For host RNA-sequencing, whole-lung RNA was extracted, depleted of host rRNA, and then prepared for sequencing. For bacterial RNA-sequencing, a pathogen-specific probe set was generated to cover coding sequences of *S. maltophilia* K279a. This pool of DNA probes was synthesized, amplified, and then reverse transcribed to create biotinylated RNA probes. Prepared cDNA libraries from the whole lung preparations were hybridized to pathogen-specific probes, and the enriched RNA population was isolated via streptavidin-bead binding. Pathogen-enriched libraries were then sequenced to obtain a bacterial transcript profile.

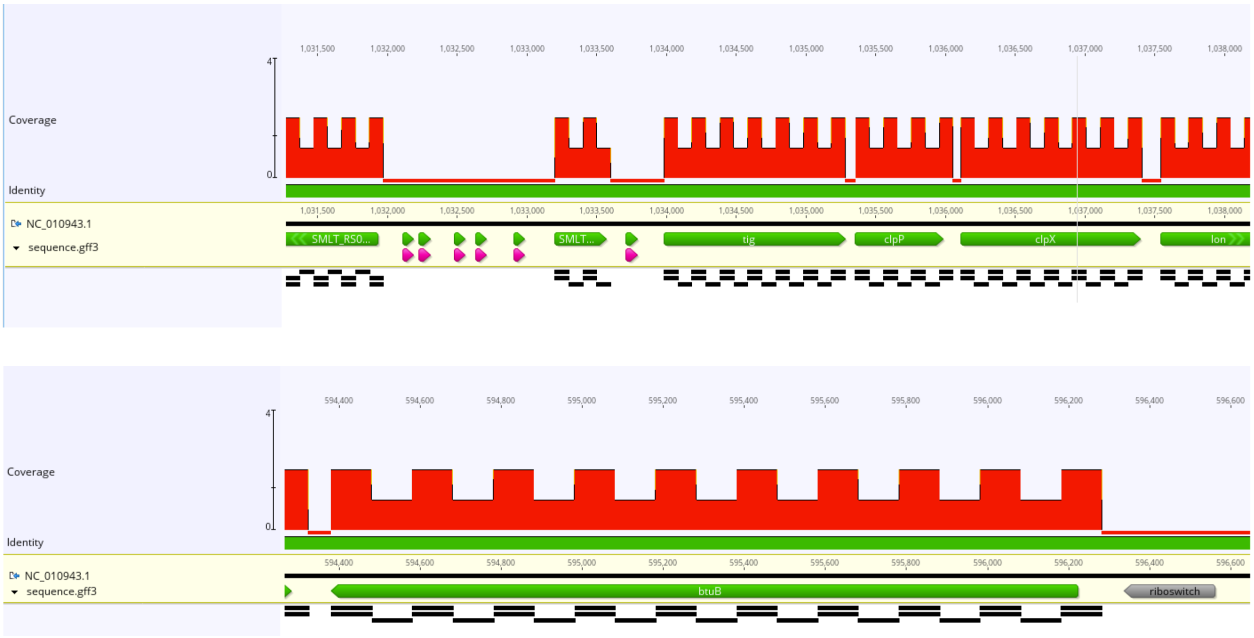

Figure S2. Alignments of PatH-Cap probes to *S. maltophilia* K279a genome.

Probes were generated to tile coding sequences in 100bp segments, with entire sense strand represented and every other 100 bp of antisense strand represented. Non-coding genome features were excluded from probe set as seen above.

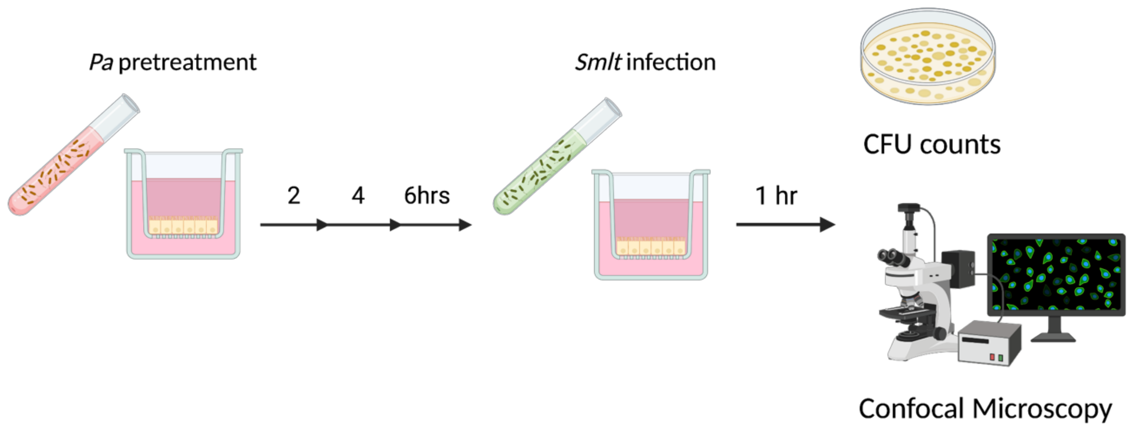

Figure S3. Infection schematic depicting the pre-treatment of with either EMEM or ~10^6 CFU of *P. aeruginosa* mPA08-31 for 2, 4, or 6 hours before the addition of ~10^6 *S. maltophilia* K279a for 1 hour.

A.

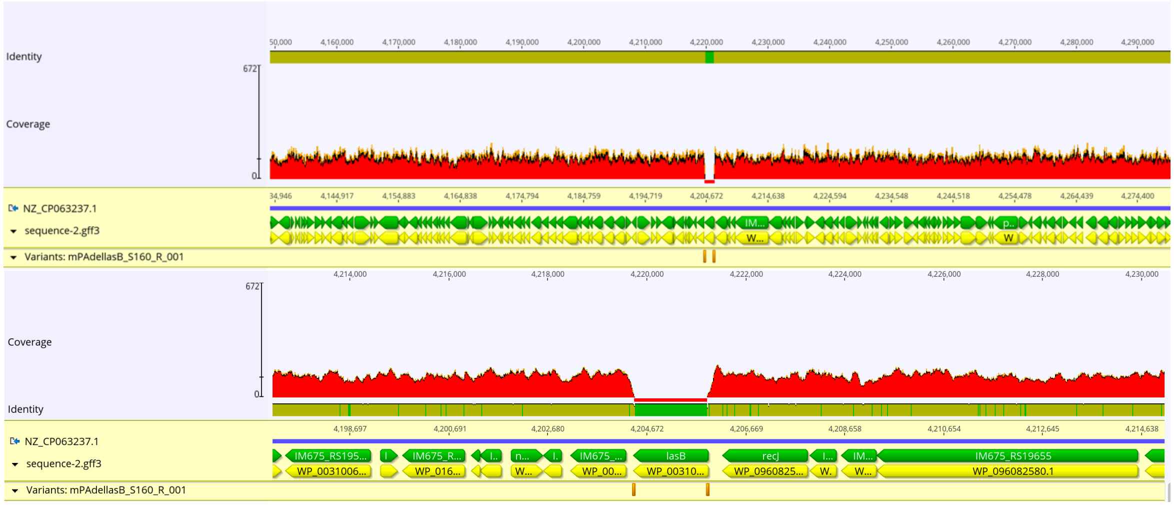

B.

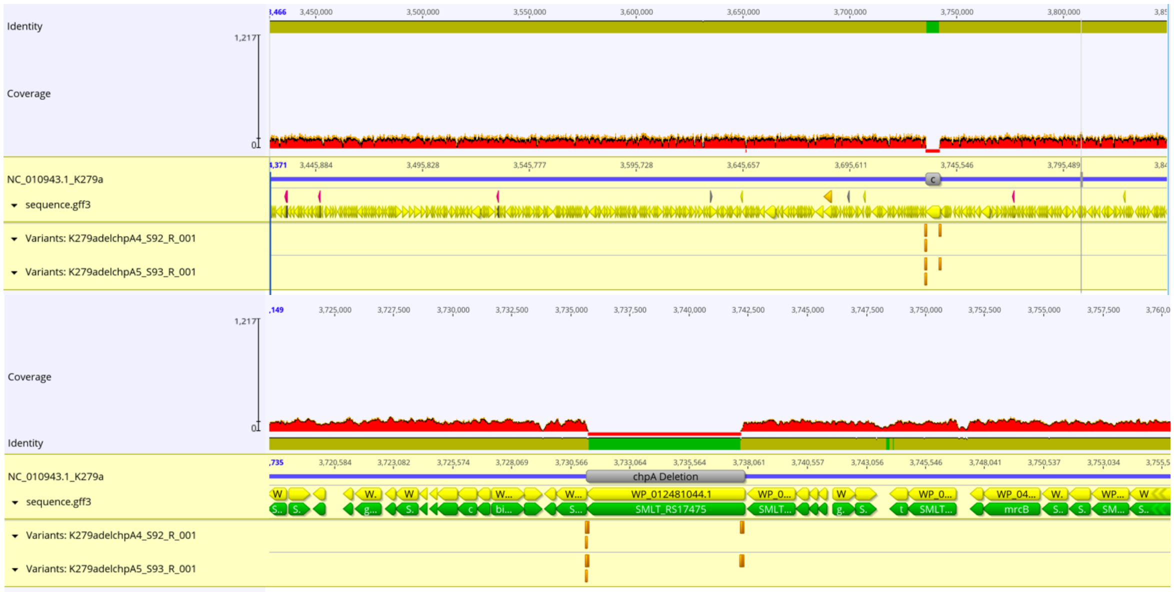

C.

***chpA* - 1**					***chpA* - 2**				
**Gene ID**	**L2FC**	***P* value**	***P* adj.**	**Gene**	**Gene ID**	**L2FC**	***P* value**	***P* adj.**	**Gene**
Smlt3668	-0.8456	0.2041	1	chpC	Smlt3668	-1.0107	0.1309	1	chpC
Smlt3669	-0.1147	0.8625	1	chpB	Smlt3669	0.0334	0.9608	1	chpB
Smlt3670	-6.3671	1.31E-14	5.75E-11	chpA	Smlt3670	-6.0799	8.62E-14	3.79E-10	chpA
Smlt3671	0.2322	0.7229	1	pilJ	Smlt3671	0.3705	0.5717	1	pilJ
Smlt3672	0.5318	0.4190	1	pilI	Smlt3672	0.5001	0.4472	1	pilI
Smlt3673	0.0946	0.8853	1	pilH	Smlt3673	0.0015	0.9985	1	pilH
Smlt3674	0.2362	0.7180	1	pilG	Smlt3674	0.1929	0.7680	1	pilG

Figure S4. Whole genome sequencing of A) *P. aeruginosa* mPA08-31 *lasB,* and B) *S. maltophilia* K279a *chpA*. For all mutants, in-frame clean deletions of the appropriate genes can be seen, with no relevant secondary mutations in surrounding genes. C) Genes downstream from *chpA* do not show differential expression between parent and *chpA* mutants.

RNA-seq round 1					
**Sample**	**Group**	**Input reads**	**Mouse % mapped**	**K279a reads mapped**	**K279a % mapped**
m22-1	K279a	22265427	73.69	253	0
m22-2	K279a	22493652	77.98	369	0
m22-3	K279a	23983260	75.88	671	0
m22-4	K279a	29492187	77.24	935	0
m22-5	K279a	19788525	79.33	1217	0.01
m22-6	Dual	24141258	72.4	636187	2.64
m22-7	Dual	25028937	77.2	1513	0.01
m22-8	Dual	28801422	78.34	1543	0.01
m22-9	Dual	23450730	77.34	649	0
m22-10	Dual	28705764	80	459	0
m22-11	mPA0831	20757985	77.23	156	0
m22-12	mPA0831	22922506	80.44	428	0
m22-13	mPA0831	27687141	77.49	358	0
m22-14	mPA0831	28341461	78.39	241	0
m22-15	mPA0831	24855211	80.38	158	0

RNAseq round 2 (PatH-Cap)					
**Sample**	**Group**	**Input reads**	**Mouse % mapped**	**K279a reads mapped**	**K279a % mapped**
m25-3K	K279a	28024276	47.54	9661463	34.48
m25-19K	K279a	31074448	55.56	4507	0.01
m25-20K	K279a	34731646	53.56	20376	0.06
m25-21K	K279a	25135729	52.95	563941	2.24
m25-23K	Dual	37830227	4.41	30333084	80.18
m25-24K	Dual	40904016	4.97	33113005	80.95
m25-25K	Dual	34263984	4.52	29489707	86.07
m25-26K	Dual	33974557	3.83	29973459	88.22

Table S1. RNA sequencing mapping statistics. Reads from sequencing were trimmed then aligned to *Mus musculus* (mm10) and *S. maltophilia* K279a genomes. Percent of total reads mapped to each genome are reported. Round 1 of sequencing was performed on whole lung RNA extractions, with depletion for rRNA from host and bacteria. Round 2 of sequencing was performed on whole lung RNA, after rRNA depletion and pathogen-specific enrichment for *S. maltophilia* K279a.

Gene ID	L2FC	*P* value	*P* adj.	Gene
Smlt3668	4.2915	1.08E-03	9.04E-03	chpC
Smlt3669	4.5002	1.20E-04	1.79E-03	chpB
Smlt3670	4.5334	5.04E-07	4.70E-05	chpA
Smlt3671	1.9556	2.08E-01	3.81E-01	pilJ
Smlt3672	6.0615	2.50E-06	1.17E-04	pilI
Smlt3673	2.8634	3.72E-02	1.21E-01	pilH
Smlt3674	6.1035	1.39E-04	1.97E-03	pilG
Smlt3756	0.9631	4.66E-01	6.47E-01	pilB
Smlt3757	6.4439	5.55E-07	4.83E-05	pilA
Smlt3758	-0.1245	9.38E-01	9.69E-01	pilA
Smlt3759	1.4679	2.81E-01	4.69E-01	pilC
Smlt3821	8.4000	1.23E-11	2.92E-08	pilQ
Smlt3822	5.6456	4.88E-04	4.98E-03	pilP
Smlt3823	6.5925	7.51E-06	2.63E-04	pilO
Smlt3824	4.0862	5.39E-04	5.34E-03	pilN
Smlt3825	3.4516	1.18E-02	5.21E-02	pilM
Smlt1089	4.9151	2.41E-03	1.63E-02	pilT
Smlt1090	2.9692	1.20E-02	5.26E-02	pilU
Smlt0612	4.8630	1.11E-03	9.14E-03	pilU

Table S2. *S. maltophilia* K279a gene expression of type 4 pilus (T4P)-associated pathways. Differential expression analysis of pilus related genes during single or dual species infection. Log fold changes indicate expression differences in dual species infections relative to single species infections. Green indicates p-values less than 0.05.

Flagellar regulation and biosynthesis operons

	GeneID	baseMean	log2FoldChai	lfcSE	stat	pvalue	padj
flgN	Smlt2321	9.51830149	1.53306512	1.93614882	0.79181161	0.42847053	NA
flgM	Smlt2320	9.69965616	1.4108005	2.56368037	0.55030281	0.5821117	NA
flgA	Smlt2319	2.53099176	-0.6004712	3.34057397	-0.1797509	0.85734812	NA
cheV	Smlt2318	9.38210884	1.29530771	3.24957641	0.39860817	NA	NA
flgB	Smlt2317	57.2199726	-0.8728489	1.46324604	-0.5965155	NA	NA
flgC	Smlt2316	49.8155532	-1.4212801	2.30748591	-0.6159431	0.53793206	0.70870603
flgD	Smlt2315	22.3450397	2.91216342	1.64717987	1.76796929	0.07706604	0.2024439
flgE	Smlt2314	27.9556551	3.29584789	1.56586815	2.1048055	0.03530824	0.11703465
flgF	Smlt2313	26.9287775	1.32970874	2.3369055	0.56900407	0.56935338	0.73063454
flgG	Smlt2312	19.6207821	2.45956674	2.36830712	1.03853369	0.29902166	0.48695727
flgH	Smlt2311	18.7396689	2.64906301	1.66099689	1.59486332	0.11074281	0.25577694
flgI	Smlt2310	15.4240354	2.30486955	1.76819946	1.30351219	0.19239996	0.36174955
flgJ	Smlt2309	11.739749	1.70194313	2.46256716	0.69112557	0.48948663	NA
flgK	Smlt2308	75.6626135	0.30294396	1.5431173	0.19631946	0.84436013	0.9200325
flgL	Smlt2307	23.8305992	1.39408921	1.87603268	0.74310497	0.45741813	0.63926314
fliC1	Smlt2306	54.8398393	3.02026489	1.71517755	1.7609051	0.07825447	0.20417396
fliC2	Smlt2305	295.925615	3.43797172	1.37440536	2.50142485	0.01236947	0.05358942
fliC3	Smlt2304	390.305996	2.9992021	1.23824915	2.42213137	0.01542977	0.06353678
fliD	Smlt2303	93.3736099	0.30205465	1.41583236	0.21334069	0.83106124	0.91375095
fliS	Smlt2302	7.7654045	-0.8797493	3.0297913	-0.2903663	NA	NA
fliE	Smlt2290	6.07674231	0.70764406	2.79145463	0.25350369	0.79987901	NA
fliF	Smlt2289	9.29875952	1.31555137	2.84687689	0.46210336	0.6440072	NA
fliG	Smlt2288	24.3158478	3.07826128	1.58641122	1.94039303	0.05233194	0.15423641
fliH	Smlt2287	6.61899967	0.78147339	3.09022459	0.25288563	0.80035659	NA
fliI	Smlt2286	31.4380276	0.4611068	2.17029383	0.21246284	0.83174596	0.91375095
fliJ	Smlt2285	10.6667403	-2.7214013	2.86796256	-0.9488971	0.34267297	NA
fliK	Smlt2284	25.492518	-0.5427848	2.24213616	-0.2420838	0.80871526	0.90081074
fliL	Smlt2283	4.77471903	0.32179641	2.6801256	0.12006766	0.90442955	NA
fliM	Smlt2282	14.4390225	2.09285135	2.05653688	1.01765807	0.30884048	0.49763924
fliN	Smlt2281	1.31771528	-1.5420759	3.34125105	-0.4615265	0.64442091	NA
fliO	Smlt2280	1.57669899	-1.2832259	3.34101907	-0.3840822	0.70091753	NA
fliP	Smlt2279	8.68844208	1.30870501	2.25060492	0.58149034	0.56091003	NA
fliQ	Smlt2278	7.0510967	0.92253659	2.78768648	0.33093269	0.74069533	NA
fliR	Smlt2277	8.12392154	1.23598141	2.15165557	0.57443274	0.565675	NA
flhB	Smlt2274	5.34671034	0.56033314	2.50595971	0.22360022	0.82306839	NA
flhA	Smlt2273	11.6133751	1.84674429	1.87036252	0.98737238	0.32346012	NA
flhF	Smlt2272	12.2836941	-1.3929103	2.47132709	-0.5636285	NA	NA
fleN	Smlt2271	7.69922978	1.06152848	2.68451545	0.39542647	0.69252818	NA
fliA	Smlt2270	5.55444883	0.5588852	2.9874472	0.18707785	0.8515996	NA
cheY	Smlt2269	5.77957443	0.68103957	2.45345928	0.2775834	0.78133218	NA
cheZ	Smlt2268	4.04089803	0.07439501	3.34029916	0.02227196	0.98223102	NA
cheA	Smlt2267	38.5066069	2.2303121	1.81909124	1.2260584	0.2201767	0.39606054
motC	Smlt2266	18.183116	2.35671477	2.33282074	1.01024255	0.3123791	0.50056221
motD	Smlt2265	39.0577151	2.26707475	1.79983298	1.25960285	0.20781267	0.38004267
rpoN	Smlt2297	40.5962904	2.49625083	1.74583954	1.42982833	0.1527663	0.31815107
	Smlt2296	36.1359539	3.69039966	1.53706549	2.40093846	0.01635309	0.06653716
fleQ	Smlt2295	114.078013	4.90860947	1.39253616	3.52494219	0.00042358	0.00450837

Fimbrial genes

	GeneID	baseMean	log2FoldChai	lfcSE	stat	pvalue	padj
smf-1	Smlt4180	83.905904	-1.908006	1.70854115	-1.1167457	0.26410307	0.44895644

Table S3. ***S. maltophilia*** **K279a gene expression of flagellar and fimbrial pathways**. Differential expression analysis of flagellar and fimbrial genes during single or dual species infection. Log fold changes indicate expression differences in dual species infections relative to single species infections. Green indicates p-values less than 0.05.

	Primer Name	Primer Sequence 5' - 3'
**Amplification of *Smlt3670* (*chpA*)**	Smlt3670 FWD	TCG GAA ATC GTG CTG GAA G
	Smlt3670 REV	GTG AAA CCA GAG GCG AAG AT
**Plasmid contruction for clean deletion of *Smlt3670* (*chpA*)**	Smlt3670_upstream_FWD	ATC CCC GGG TAC CGA GCT CGG GTG CGC CGG TGG CGC ATA CAG G
	Smlt3670_upstream_REV	CCG GCG CAC CAG CCC AGG CGG CCC CGG C
	Smlt3670_downstream_FWD	CGC CTG GGC TGG TGC GCC GGT GGC GCA TAC AGG
	Smlt3670_downstream_REV	CAG CTA TGA CCA TGA TTA CGA GCC CAG GCG GCC CCG GC
**Amplification of *lasB***	lasB FWD	ACTCGATGAAACGGGTGATG
	lasB REV	CCTTCTACCCGAAGGACTGATA
**Plasmid contruction for clean deletion of *lasB***	lasB_upstream_FWD	ATC CCC GGG TAC CGA GCT CGG TCC TAC CGG GAT TTC CGC ATT CGC CG
	lasB_upstream_REV	ACT GAA CAA GGC TCG GTG GCC CCG GCC G
	lasB_downstream_FWD	GCC ACC GAG CCT TGT TCA GTT CTC CTG GTT TTT TCA GGC C
	lasB_downstream_REV	CAG CTA TGA CCA TGA TTA CGG AGG CGC TGG CGC AGC TC
**Amplification of cDNA library (maintains barcoding) - PatH-Cap**	NEB hyb primer FWD	AAT GAT ACG GCG ACC ACC GAG ATC TAC ACT CTT TCC CTA CAC GAC GCT CTT CCG ATC T/3ddC/
	NEB hyb primer REV	CAA GCA GAA GAC GGC ATA CGA GAT NNN NNN NNG TGA CTG GAG TTC AGA CGT GTG CTC TTC CGA TCT /3ddC/
**Amplification of cDNA library (no barcoding) - PatH-Cap**	P5 primer	AAT GAT ACG GCG ACC ACC GAG ATC TAC ACT CTT TCC CTA CAC GAC GCT CTT CCG ATC
	P7 primer	CAA GCA GAA GAC GGC ATA CGA GAT

Table S4. Primers used in present study. *lasB* and *chpA* primers were used for the construction of plasmids for clean deletion via homologous recombination.
